# The applications of functional materials-based nano-formulations in the prevention, diagnosis and treatment of chronic inflammation-related diseases

**DOI:** 10.3389/fphar.2023.1222642

**Published:** 2023-08-01

**Authors:** Jingjing Wang, Rui Ni, Tingting Jiang, Dan Peng, Yue Ming, Hongjuan Cui, Yao Liu

**Affiliations:** ^1^ Department of pharmacy, Daping Hospital, Army Medical University, Chongqing, China; ^2^ Medical Research Institute, Southwest University, Chongqing, China

**Keywords:** nanotechnology, chronic inflammation, prevention, diagnosis, treatment

## Abstract

Chronic inflammation, in general, refers to systemic immune abnormalities most often caused by the environment or lifestyle, which is the basis for various skin diseases, autoimmune diseases, cardiovascular diseases, liver diseases, digestive diseases, cancer, and so on. Therapeutic strategies have focused on immunosuppression and anti-inflammation, but conventional approaches have been poor in enhancing the substantive therapeutic effect of drugs. Nanomaterials continue to attract attention for their high flexibility, durability and simplicity of preparation, as well as high profitability. Nanotechnology is used in various areas of clinical medicine, such as medical diagnosis, monitoring and treatment. However, some related problems cannot be ignored, including various cytotoxic and worsening inflammation caused by the nanomaterials themselves. This paper provides an overview of functional nanomaterial formulations for the prevention, diagnosis and treatment of chronic inflammation-related diseases, with the intention of providing some reference for the enhancement and optimization of existing therapeutic approaches.

## 1 Introduction

Inflammation is a response involving immune and non-immune cell activation that promotes tissue repair and protects the host from a variety of infections, toxins and so on. The main categories are acute inflammation and chronic inflammation. Acute inflammation is defined as inflammation that occurs in the short term through interactions between innate immune cells and pathogens or in response to harmful stimuli during cellular stress or injury, while chronic inflammation usually persists through the latter and eventually damages tissues and organs to cause chronic inflammatory disease (Furman et al., 2019; Reichardt et al., 2021; Shah et al., 2023). The inflammatory response process can be divided into 1) damage to the body’s tissues and cells, 2) recognition of damage factors and tissue necrosis and production of inflammatory mediators, 3) dilution, neutralization, killing and removal of harmful substances, 4) abatement and termination of the inflammatory response, and 5) repair of damaged tissues (Chen et al., 2018; Schett and Neurath, 2018; Reichardt et al., 2021). Targeted modulation of inflammation is a common therapeutic strategy, but the low bioavailability, short half-life, and non-specificity of many drugs contribute to their less-than-optimal therapeutic efficacy. Therefore, it is crucial to develop more effective therapeutic approaches to address these issues (Song et al., 2023).

Nanotechnology aims to manipulate the unique properties of matter at the nanoscale, which can be observed instrumentally to exhibit unique characteristics and develop new capabilities with potential applications, which is one of the main reasons why they have various applications across all fields of medicine (Xu et al., 2011; Aguilar, 2012; You and Bonner, 2020). Recent advances in nanotechnology and nanofabrication have shown that various nanostructures and devices are being used in various applications such as medical diagnostics, monitoring and therapeutic applications (Li P. et al., 2021; Ayodele et al., 2021; Baek et al., 2021; Zhu et al., 2021).

The inflammatory response involves many cells and chemical modulators for protection from infection (Johnson et al., 2022). In most cases, it is beneficial as an automatic defense response of the body. However, inflammatory conditions are also harmful to the organism, such as attacks on the body’s tissues (Jin et al., 2018). Common inflammatory disorders include systemic lupus erythematosus (SLE), arthritis, tonsillitis, pneumonia, gastritis, enteritis and so on (Roe, 2021). Targeting inflammation, therefore, offers a promising solution for the diagnosis and treatment of these diseases. Identifying which cell types are key to their pathogenesis will allow drugs to eventually penetrate or cross endothelial cells by binding nanocarriers to key targets, providing a more accurate and efficient therapeutic effect (Howard et al., 2014). Recently, research has shown that reproducible, enhanced bionanoparticles have the potential to improve the treatment of inflammatory diseases through targeted nano-delivery (Jin et al., 2018; Wang et al., 2020; Zinger et al., 2021; Khatoon et al., 2022). This article provides an overview of the use of functional nanomaterials and agents in the prevention, diagnosis, and treatment of chronic inflammation-related diseases, to optimize existing therapeutic approaches and inform more effective treatment options.

## 2 Chronic inflammatory diseases and nanomaterials

**FIGURE 1:**
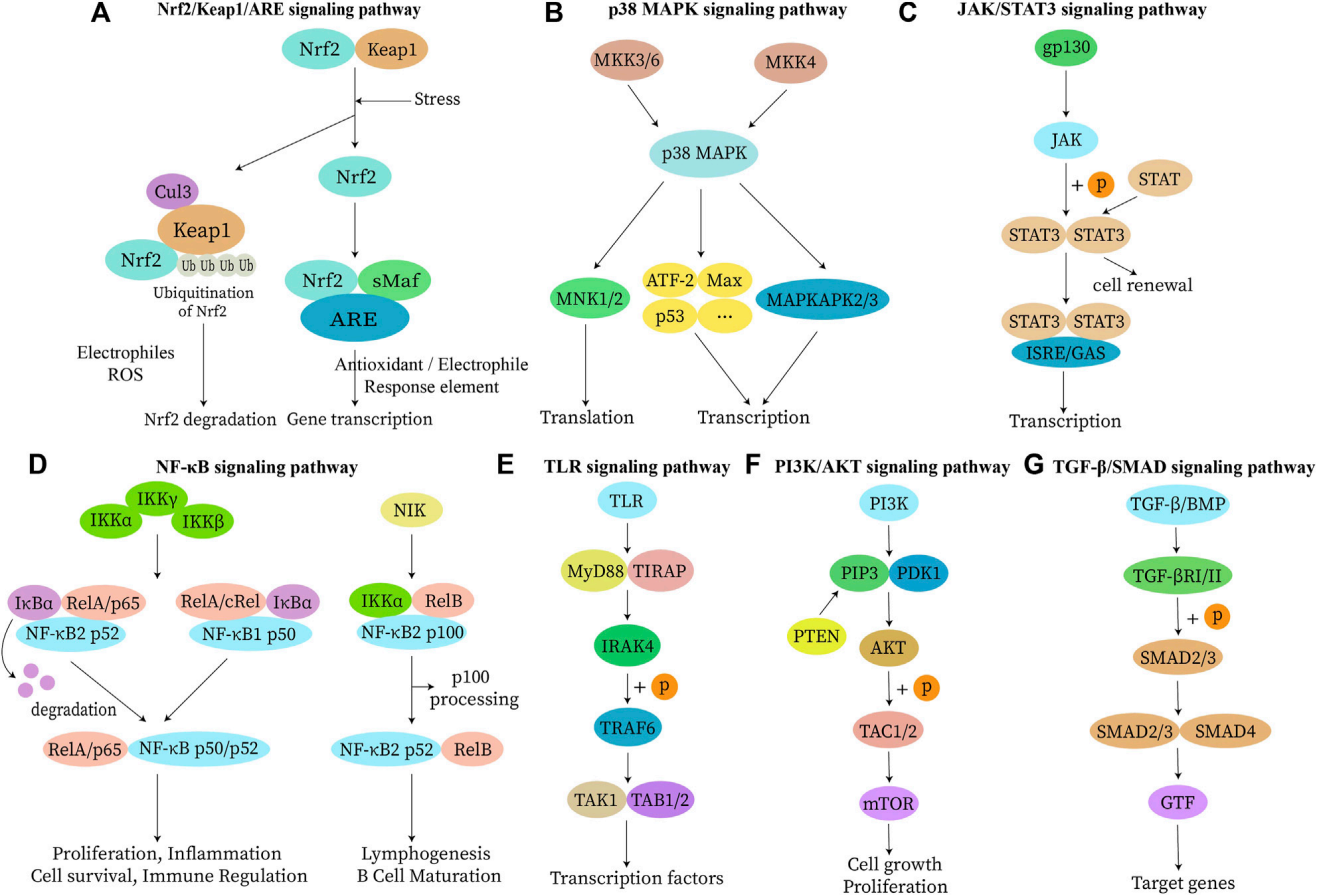
Inflammation-related signaling pathways. Continued inflammatory response promotes the development of various diseases when uncontrolled inflammation occurs *in vivo*. Immune cells can change their phenotypic and functional characteristics in response to environmental changes. Therefore, a knowledge of the inflammation-related action mechanisms not only improves therapeutic efficacy but also facilitates drug development. The inflammatory response activates kinases through signal transduction by B cell receptors and T lymphocyte receptors. Activated kinases initiate an intracellular signaling cascade by enhancing signaling through phosphorylation of downstream junction proteins. **(A)** Nrf2 and Keap1 are degraded by ubiquitination or activation, resulting in the release and translocation of Nrf2 into the nucleus. There, it binds to ARE to activate downstream gene transcription and translation of related proteins, enabling the performance of physiological functions. **(B)** MKK can be activated by phosphorylation of MAPKKK or direct activation in response to stimuli such as apoptosis. p38 MAPK is involved in the regulation of MNK 1, MNK 2, MAPKAPK 2, MAPKAPK 3, and several other transcription factors, including ATF-2, Stat1, Max, and p53. **(C)** IL-6 (gp130) binding causes dimerization of the receptor while activating the receptor-bound JAK protein. Activated JAK phosphorylates the receptor and itself and becomes a binding site for STAT proteins and junction proteins, linking the receptor to other pathways such as MAPK and PI3K/AKT. **(D)** Upon receiving stimulation, activation of IκB kinase triggers IκB protein degradation and consequent release of NF-κB dimer. The released NF-κB dimer is further activated via post-translational modification and subsequently transported into the nucleus where it binds to its target genes, promoting transcription. **(E)** TLR detects pathogen-associated molecular patterns and a conserved family of pattern recognition receptors in microbial pathogens. This leads to amplification of intracellular signal transduction regulators such as MyD88, IRAKs, and TRAF6, and activation of MAPK and NF-κB signaling pathways to induce inflammation. **(F)** PI3K binding to EGFR can modify AKT protein structure and control downstream substrate activity through phosphorylation, either activating or inhibiting it. Additionally, PI3K can activate IKK, which directly affects the NF-κB pathway and regulates cell proliferation, differentiation, apoptosis, and migration. **(G)** Dimeric TGF-β ligands bind to their corresponding type II and type I receptors on the cell membrane, leading to phosphorylation of the type I receptor by the type II receptor and subsequent activation of its kinase activity. The activated type I receptor then recruits and stimulates downstream SMAD proteins as transcription factors to regulate gene expression. Abbreviations: Nrf2, nuclear factor E2-related factor 2; Keap 1, kelch-like ECH associated protein 1; ARE, antioxidant response element; Cul3, cullin-based E3 ligase; sMaf, small Maf proteins; p38 MAPK, mitogen-activated protein kinase p38 antibody; MKK, p38 MAPK kinase; MNK, MAPK signaling integrated kinase; ATF-2, activating transcription factor 2; Max, MYC associated factor X; MAPKAPK, MAPK-activated protein kinase; JAK, janus kinase; STAT, signal transducers and activators of transcription; gp130 (IL-6), interleukin 6; ISRE, interferon stimulated response element; GAS, growth arrest-specific transcripts; NF-κB, nuclear factor κB; IKK, inhibitor of kappaB kinase; IκBα, inhibitor kappa B alpha; Rel, REL proto-oncogene; NIK, NF-κB-inducing kinase; TLR, toll-like receptor; Myd88, myeloid differentiation primary response protein 88; TIRAP, TIR domain-containing adapter protein; IRAK4, IL-1 receptor-associated kinase 4; TRAF6, TNF receptor associated factor 6; TAK1, transforming growth factor-beta-activated kinase 1; TAB1/2, MAP3K7 binding protein 1/2; PI3K, phosphatidylinositol-3-kinase; PIP3, phosphatidylinositol-3,4,5-bisphosphate; PDK1, 3-phosphoinositide-dependent protein kinase 1; AKT, serine/threonine kinase; TAC, tachykinin precursor gene; mTOR, mammalian target of rapamycin; TGF-β, transforming growth factor β; SMAD, *drosophila* mothers against decapentaplegic protein; BMP, bone morphogenetic protein; TGF-βR, TGF-β receptor; GTF, glucose tolerance factor.

**FIGURE 2:**
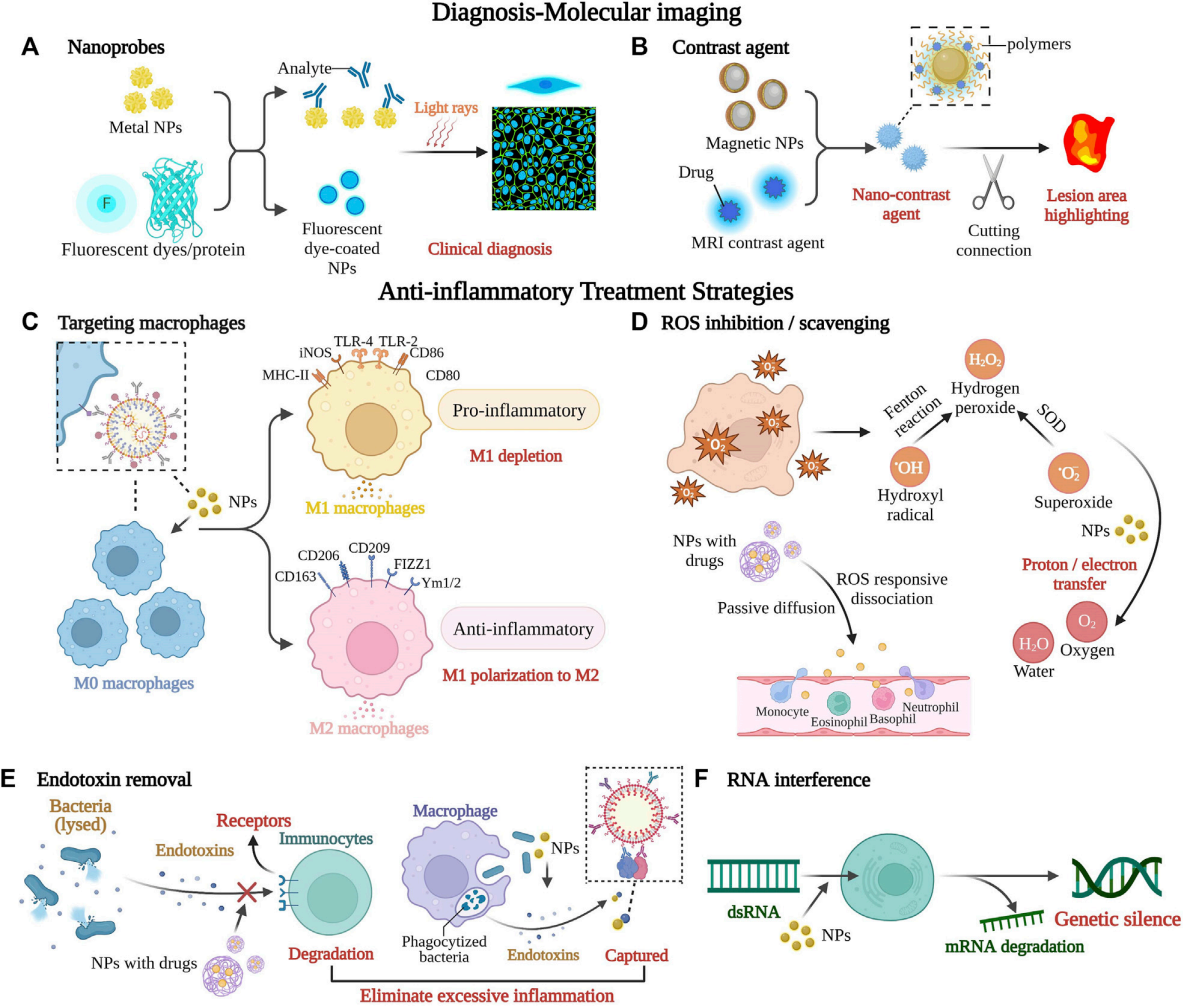
Major strategies for diagnosing and treating inflammatory diseases with nano-formulations. Nano-formulations can improve molecular imaging accuracy to aid diagnosis and identification of disease areas, as well as modulate the inflammatory microenvironment at various stages of inflammation development by removing unfavorable molecules, blocking immune cells, and facilitating the precise delivery and release of immune drugs, among other strategies. They have enormous potential to intervene in inflammatory disease processes and improve therapeutic efficacy. **(A)** Nanomaterials coated with fluorescent dyes or coupled with fluorescent proteins make extremely sensitive nano-biochemical sensors for early diagnosis. **(B)** The nanocontrast agent is based on magnetic resonance technology and consists mainly of two magnetic materials, including magnetic nanoparticles and paramagnetic MRI contrast agent, and the brightness of MRI is changed by the distance between the two, with a critical distance of 7 nm. **(C)** Macrophages stimulated by interferon γ or interleukin l4 will differentiate into M1 macrophages that promote inflammation or M2 macrophages that suppress inflammation. Inflammatory disease treatment should promote the depletion of M1 macrophages and/or polarization to M2 macrophages. **(D)** ROS inhibition/scavenging plays an important role as one of the main approaches to reduce oxidative stress. Inorganic nanomaterials can convert ROS to oxygen and water, while the organic fraction can eliminate ROS by proton or electron transfer. **(E)** Endotoxin is a collective term for the toxic substances present in Gram-negative bacteria. Nanoparticles with similar activity to specific enzymes can hydrolyze lipopolysaccharides on the surface of endotoxins, leading to their inactivity and eventual degradation, as well as capture endotoxins and inhibit excessive inflammation. **(F)** Targeted delivery of RNA by nanoparticles leads to efficient gene silencing at mRNA levels, which means a significant decrease in protein expression. This figure was created with BioRender.com. Abbreviations: NPs, nanoparticles; MRI, magnetic resonance imaging; MHC-II, major histocompatibility complex class Ⅱ; iNOS, inducible nitric oxide synthase; TLR, toll-like receptor; CD, cluster of differentiation; FIZZ1, resistin-like molecule alpha 1; Ym, chitinase-like protein; ROS, reactive oxygen species; dsRNA, double-stranded ribonucleic acid; mRNA, messenger ribonucleic acid.

When the inflammatory response is inadequate or excessive, it can disrupt the body’s immune system balance and lead to the development of chronic inflammatory diseases if it is not addressed on time (Locati et al., 2020). Chronic inflammation responds variably to different pathogens and adapts flexibly to different microenvironments, resulting in a diversity of responses (Schett and Neurath, 2018). It is beneficial for cancer and triggers autoimmune diseases in severe cases (Roe, 2021). Therefore, identifying the cause of inflammation and improving treatment efficiency is essential for the future of healthcare. Nanomaterials play a non-negligible role in it. Current studies involve nanomaterials themselves or use them as delivery vehicles to achieve excessive inflammation removal by targeting specific cells, improving drug delivery efficiency and bioavailability (Zinger et al., 2021; Khatoon et al., 2022). Chronic inflammation-related signaling pathways and associated nano-formulations are shown in Figure 1 and Table 1. Major strategies for diagnosing and treating inflammatory diseases with nano-formulations and their specific applications are listed in Figure 2 and Supplementary Table S2.

**TABLE 1:** Classification and application of nanomaterial formulations based on inflammation-related signaling pathways.

Signaling pathways	Related cytokines	Nano-formulations	References
Nrf2/Keap1/ARE	RXRα, Keap3, Keap1-Cul3-RBX1 E3 ligase complex, sMaf, AREs, EpRE, β-TrCP, etc.	natural carrier NPs, inorganic NPs, etc.	Manna et al. (2019), Gu et al. (2021)
P38 MAPK	TRAF2, MKK3, ATF-2, HSP27, MAPKAPK2, MAPKAPK3, MNK1, MNK2, etc.	inorganic NPs, polymeric NPs, etc.	Chiang et al. (2021), Hu et al. (2021)
JAK/STAT3	G-CSF, EPO, TPO, EGFR, etc.	polymeric NPs, inorganic NPs, etc.	Nag et al. (2020), Lin et al. (2022)
NF-κB	p52/p100, p50/p105, c-Rel, RelA/p65, RelB, IκBα, TNFR, IL1β, TNF-α, IL-6, etc.	liposomal NPs, inorganic NPs, polymeric NPs, etc.	Lu et al. (2020), Kim et al. (2021), Liu et al. (2023)
TLR	TIR, MyD88, TIRAP, IRAK, TRAF6, etc.	liposomal NPs, inorganic NPs, etc.	Zhang et al. (2021b), Shi et al. (2022)
PI3K/AKT	p85/p110, PIP2, PDK1, AKT, TSC1/2, PTEN, etc.	polymeric NPs, inorganic NPs, etc.	Li et al. (2022b), Huang et al. (2022)
TGF-β/SMAD	TGF-β, TGF-βRI, TGF-βRII, SMAD, BMP, GDF, etc.	inorganic NPs, polymeric NPs, etc.	Alomari et al. (2020), Kim et al. (2021)

Nrf2, nuclear factor E2-related factor 2; Keap 1, kelch-like ECH, associated protein 1; ARE, antioxidant response element; RXRα, retinoic X receptor α; Cul3, cullin-based E3 ligase; RBX1, ring box protein 1; sMaf, small Maf proteins; AREs, antioxidant response elements; EpRE, electrophile response element; β-TrCP, beta-transducin repeats-containing protein; NPs, nanoparticles; p38 MAPK, mitogen-activated protein kinase p38 antibody; TRAF2, tumor necrosis factor receptor-associated factor 2; MKK3, p38 MAPK, kinase 3; ATF-2, activating transcription factor 2; HSP27, heat shock protein 27; MAPKAPK, MAPK-activated protein kinase; MNK, MAPK, signaling integrated kinase; JAK, janus kinase; STAT3, signal transducers and activators of transcription 3; G-CSF, granulocyte colony-stimulating factor; EPO, erythropoietin; TPO, thrombopoietin; EGFR, epidermal growth factor receptor; NF-κB, nuclear factor κB; Rel, REL, proto-oncogene; IκBα, inhibitor kappa B alpha; TNFR, tumor necrosis factor receptor; IL, interleukin; TNF-α, tumor necrosis factor-alpha; TLR, toll-like receptor; TIR, toll-interleukin 1 receptor; Myd88, myeloid differentiation primary response protein 88; TIRAP, TIR, domain-containing adapter protein; IRAK, IL-1, receptor-associated kinase; TRAF6, TNF, receptor associated factor 6; PI3K, phosphatidylinositol-3-kinase; AKT, serine/threonine kinase; PIP2, phosphatidylinositol-4, 5-bisphosphate; PDK1, 3-phosphoinositide-dependent protein kinase 1; TSC, tuberous sclerosis complex; PTEN, protein tyrosine phosphatase; TGF-β, transforming growth factor beta; SMAD, *drosophila* mothers against decapentaplegic protein; TGF-βR, TGF-β, receptor; BMP, bone morphogenetic protein; GDF, growth differentiation factors.

### 2.1 Inflammation in skin diseases

Most inflammatory skin diseases have a chronic recurrent course, such as chronic plaque psoriasis (psoriasis vulgaris) and atopic dermatitis, which have an inflammatory component in the pathological model (Boehncke and Schön, 2015; Boehncke and Brembilla, 2018; Guttman-Yassky et al., 2018). However, its safety and efficacy are greatly constrained by the drug’s limited accessibility to deeper skin layers and adverse reactions (Guttman-Yassky et al., 2018; Cláudia Paiva-Santos et al., 2022). This limitation can be improved by using nanoparticles (NPs) that enhance the penetration of bioactive compounds into deeper layers of the skin, providing sustained drug release and targeting specific cells with long-lasting effects (Raszewska-Famielec and Flieger, 2022).

Nanocarriers can penetrate the stratum corneum without restriction as well as the skin through various routes. Nanotechnology-based carriers improve durability, which protects the drug from degradation and improves the effectiveness of psoriasis treatment (Parveen et al., 2022). Elmowafy E et al. investigated a novel formulation of topically directed nanovesicles, materialized and characterized tazarotene-loaded fluidized spanlastic nanovesicles (the combination of spanlastics and penetration enhancer vesicles). It was clinically evaluated as superior to the commercial product Acnitaz in dermoscopic imaging and morphological analysis of psoriatic lesions, which shows a large potential application (Elmowafy et al., 2019). Curcumin, as a natural anti-inflammatory compound that induces the expression and production of inflammatory cytokines, gradually combines with nanomaterials to have a multifaceted role in the treatment and management of chronic diseases (Mollazadeh et al., 2019; Tagde et al., 2021). To assist avelic acid in the treatment of moderate to severe psoriasis patients, curcumin loaded poly (vinylpyrrolidone)-30 NPs of 785.50 ± 30.16 nm average diameter were developed and used in clinical studies. It enhanced the solubility of curcumin and serves as an effective adjunctive therapy for controlling serum cholesterol levels and improving lipid and serum levels in psoriasis patients (Bilia et al., 2018).

In addition, various types of dermatitis are also a very disturbing part of complex skin disorders. A randomized controlled trial found that creams containing vitamin E NPs provided a protective effect against radiation dermatitis, demonstrating the potential application of nano-encapsulated antioxidants (Queiroz Schmidt et al., 2022). An *in vitro* immunoassay has been proposed based on the specific memory T-cell properties for circulating allergens, encapsulating hydrophobic allergens into nanoparticle (NP) carriers that skip the serum analysis, opening up a new avenue for *in vitro* immunobiological diagnosis of allergic contact dermatitis (Cortial et al., 2015). HC-HT CSNP AQ cream is chitosan-based pentasodium tripolyphosphate NPs of <250 nm loaded with hydrocortisone and hydroxytyrosol, anti-microbial and anti-oxidant agents, which are safe, well tolerated, non-toxic for application in therapeutic regimens (Siddique et al., 2019). Controlling the speed and performing overhead mixing by using the spinning disc technique has great potential for the large-scale preparation of NPs that are physiochemically identical to laboratory-scale ones. On the other hand, due to the special accessibility of the skin, pilot studies have been carried out on relevant nanofabrics. The results indicate that nanotextiles (100% nano-polyester) have shown good potential in wet wrap therapy for patients with moderate to severe eczema as effectively as conventional mucilage (He et al., 2020).

Research on nanomaterials related to chronic inflammatory skin diseases is relatively scarce compared with other diseases, especially in terms of prevention. As a currently incurable disease, it can also trigger other reactions and should receive more attention. In contrast, in addition to the use of nanomaterials for precise and targeted drug delivery, for example, customized nanofabrics can lead to better patient care and acceptance, which is one of the possible directions for further development of complementary therapies.

### 2.2 Inflammation in rheumatoid arthritis and systemic lupus erythematosus

Rheumatoid arthritis (RA) is one of the most common chronic joint diseases, causing chronic synovitis and bony joint destruction, as well as other diseases in the body that ultimately lead to increased mortality (Scott et al., 2010; Pieringer et al., 2014; Zhao et al., 2022). SLE is also a systemic autoimmune disease with a complex etiology, leading to increased bone loss and fracture development, and treatment requires suppression of disease activity and prevention of irreversible organ damage (Bultink, 2018). Both diseases are inextricably linked to inflammation and its associated cytokines.

The use of NPs has been found to directly target T cells and significantly improve lupus-related pathology through various mechanisms, such as induction and amplification of CD4^+^ and CD8^+^ Tregs, but currently remains in animal models before reaching the clinical stage (Esensten et al., 2018; Li et al., 2018; Moorman et al., 2021). In RA, however, the goal is to convert dendritic cells in the disease site from immunogenic to tolerogenic and convert local macrophages from inflammatory to resistant cells through precisely targeted nanomaterial properties s for the relief and treatment of SLE (Esensten et al., 2018; Moorman et al., 2021).

Inflammation-induced bone erosion can lead to the development of RA. Previous studies have suggested that ultra-small nanoclusters, such as Au clusters, may be novel nanomedicine candidates for the treatment of RA (Gao et al., 2019). The gold (Au) cluster strongly inhibits the receptor activator of nuclear factor κB (NF-κB) ligand-induced osteoclast formation *in vitro* by suppressing the activation of the NF-κB pathway, offering a new therapeutic strategy (Yuan et al., 2019). Plant virus NPs are not capable of replicating in mammals, making them safer to use (Koudelka et al., 2015; Zampieri et al., 2020). Zampieri R et al. designed a tomato bushy stunt virus NP containing a 30 nm icosahedral shell. The repetitive administration caused downregulation of T-cell populations like Th1, Th17 and Treg cells and pro-inflammatory cytokines including tumor necrosis factor-alpha (TNF-α), interleukin 1β (IL-1β), IL-17 and interferon γ (IFN-γ). Giving tomato bushy virus NPs expressing pFADK2 and pLIP1 (immunodominant peptides associated with rheumatoid factor) even after the onset of RA could lead to complete remission of arthritic symptoms and achieved exceptional therapeutic effects (Zampieri et al., 2020).

Methotrexate is a clinical treatment options for RA, which can provide temporary relief from systemic symptoms. It has been noted that short and low doses of alpha-ketoglutarate-based polymeric α-ketoglutaric acid NPs and Methotrexate, with a particle size of 273.7 ± 62.6 nm, can offer long-term alleviation of RA symptoms. Treatment with a combination of short and low doses of them can attenuate the systemic symptoms in collagen-induced arthritis mice, driven by the downregulation of T helper cells (Th) 17 bc2-specific antigenic responses and enhancement of Th2-type T cell responses (Mangal et al., 2022). Meanwhile, chitosan NPs have also yielded noteworthy research results (Yegireddy et al., 2022; Yang et al., 2023). The authors designed a chitosan-coated meloxicam NP for sustained drug delivery and conducted experiments in Wistar rats, indicating enhanced drug delivery, rapid onset of action, and prolonged activity of MLX (Yegireddy et al., 2022). Yang L et al. have prepared a novel bionic lubricant diclofenac sodium-loaded chitosan-chondroitin sulfate NPs of 132.2 nm with dual functions of water lubrication and anti-inflammatory properties. Its ability to efficiently deliver drugs and sustain DS release further protects chondrocytes from damage (Yang et al., 2023).

Chitosan-based nanomaterials are increasingly being applied and there appears to be great promise for appropriate clinical studies to develop their potential applications in therapy. Notably, nanovaccines are also emerging as another hot topic of research and have achieved some positive results in anti-RA, making them ideal candidates for anti-RA drugs (Zhang N. et al., 2022).

Severe manifestations of SLE can lead to lupus nephritis, although the exact mechanisms remain elusive (Olivares et al., 2018). It has been reported that P2Ns-gambogic acid encapsulated cyclosporine A has the potential to treat SLE by effectively enhancing drug delivery and enhancing the targeting of lymphoid tissue (Ganugula et al., 2020). Chemiluminescent enzyme immunoassay is increasingly being used as a method that provides significantly shorter analysis times compared with enzyme-linked immunosorbent assay (Obara et al., 2018). Zhou ZR et al. proposed an extracellular vesicle biosensor based on plasma Au NPs-embedded polydopamine substrate of 52 nm to detect extracellular vesicle biomarkers in the serum and urine of SLE patients. The results suggest that the detection of sialic acid, miRNA-146a and PD-1 expression levels has great potential in the diagnosis and evaluation of SLE (Zhou et al., 2022). Additionally, other researcher has developed a quantum dots and acrylonitrile-butadiene-styrene reporting system that allows direct detection of super oligomeric mannose-binding lectin particles in plasma, which found that its formation was stabilized *in vitro* by dsDNA and increased in plasma of SLE patients (Juul-Madsen et al., 2021).

Mycophenolate mofetil is a commonly used immunosuppressant for SLE. However, its clinical efficacy is hampered by poor biodistribution in cells and tissues and its short half-life (Broen and van Laar, 2020; Trevisonno et al., 2023). Dextran mycophenolic acid-based NPs significantly improved the pharmacokinetics of mycophenolate mofetil/mycophenolic acid and alleviated SLE by promoting local m2-like macrophage polarization, as evidenced by reduced renal injury, pathological signs, and lower urinary protein-creatinine ratio (Jiang et al., 2022). In addition, inhibition of excessive mTOR signaling activation by rapamycin can be used as a complementary modality to assist in restoring immune homeostasis. Zhang et al. designed a rapamycin-encapsulated ICOS/CD40L-bispecific NPs for multitarget therapy in a disease-specific manner. They demonstrated that the NPs could selectively target SLE Th cells and effectively inhibit Th-B-cell mutual activation, promote bystander Treg cells, and significantly alleviate SLE progression with a favorable safety profile (Zhang et al., 2022a).

Combining these studies, it is easy to see that the combination of nanomaterials and drugs not only modulates the immune system to induce tolerance for preventive and therapeutic effects, but also provides new perspectives on the diagnosis and treatment of SLE. In future research, it is possible to focus on combined therapeutic strategies to more specifically prevent or block abnormal immune system responses.

### 2.3 Inflammation in cardiovascular diseases

Endothelial cells on the surface of the vascular lumen are key targets for the treatment of inflammatory, cardiovascular and other diseases. Free drugs are cleared from the blood and diffuse into non-target tissues, including the brain. In these tissues, the drug may cause adverse reactions, and reducing diffusion into non-target tissues can effectively inhibit these reactions (Howard et al., 2014). Atherosclerotic vascular disease places a huge burden on patients in terms of mortality and morbidity, with several potential risk factors present that have been assumed or not yet identified. Relevant studies have shown a link between infection, inflammation and atherosclerosis (Lim et al., 2019).

Heart failure is a serious clinical and public health problem, and the demand for effective treatment has not yet been met. Liu C et al. synthesized a reactive oxygen species (ROS) scavenging material (TPCD) and processed it into NPs (TPCD NP) with an average diameter of 101 nm to construct a multistage targeted nanotherapeutic triphenylphosphine-decorated TPCD NP to evaluates its efficacy in mice with Doxorubicin-induced cardiomyopathy *in vivo*. The results showed that it could attenuate Doxorubicin-induced oxidative stress and cellular damage by internalizing cardiomyocytes and scavenging excess ROS, demonstrating that non-invasive inhalation delivery of nanotherapeutics can prevent heart failure (Liu C. et al., 2021).

Prevention of cardiovascular disease is vital, but early diagnosis and treatment are even more crucial. A recent study has designed an all-nano-fiber optic force-acoustic sensor by using a durable, ultra-thin (2.5 µm) nanofiber electrode layer, which shows extremely high sensitivity in the low-frequency region (<500 Hz), high mechanical robustness and bending stability for long-term cardiac monitoring (Nayeem et al., 2020). The delivery of proangiogenic hepatocyte growth factor and insulin-like growth factor (IGF-1) using 50–100 nm spherical alginate-sulfate NPs improved left ventricular repair with a significant increase in ejection fraction and myocardial remodeling. The feasibility and effectiveness of growth factor delivery system were demonstrated, offering the prospect of innovative therapeutics (Wu et al., 2023). NP inhalation drug delivery is a safe and effective strategy for the targeted treatment of heart disease, and drugs for heart failure and other heart diseases are still being discovered.

NPs can specifically deliver diagnostic and therapeutic drugs to modulate atherosclerotic pathology (Zhang X. et al., 2023). Macrophages, the center of vascular inflammation and vascular lesion growth in atherosclerosis, are important targets for the diagnosis and treatment of diseases (Boada et al., 2020; Chen et al., 2022). Systemic rapamycin may reduce atherosclerotic plaque development, although it has been associated with many adverse effects due to off-target effects. Validation studies revealed that rapamycin-coated biomimetic NPs (leukosomes) with a diameter of 108 ± 2.3 nm were able to inhibit macrophage proliferation in aortic tissue through their specific targeting properties, associated with reduced levels of monocyte chemoattractant protein-87 and IL-b1. It was able to effectively reduce inflammation in atherosclerotic mice and enhance therapeutic efficacy while maintaining a good safety profile (Boada et al., 2020). Molecular imaging facilitates the visualization of high-risk atherosclerotic plaques and assists in the development of therapeutic drugs (Chen et al., 2022). Poon C et al. investigated the design of a mixed metal oxide-peptide amphiphilic micelle capable of enhanced thrombi binding with potential magnetic resonance imaging in atherosclerotic plaque thrombi, with potential as an early diagnostic method for atherosclerotic thrombi (Poon et al., 2018).

IGF-1 plays an important role in growth, metabolism and homeostasis, and increasing its circulation may reduce the atherosclerotic burden. However, overactive IGF-1 can remain in tissues causing adverse effects, resulting in a very low circulating level. A hydrogel of self-assembled naproxen-modified peptide was used to mimic the biological activity of IGF-1, which performed positively in apoE^−/−^ mice and inhibited atherosclerosis by enhancing plaque stability and significantly reducing lesion size (Shang et al., 2020). Notably, kaempferol has potential anti-inflammatory effects as a novel anti-inflammatory drug candidate, which has rarely been reported. A macrophage-mimetic kaempferol delivery platform of ∼200 nm mimicking macrophages was constructed and its action mechanism was correlated with the blocking of ROS/NF-κB signaling pathway. It significantly reduced the macrophage proliferation inflammation while decreasing TNF-α and increasing IL-10 levels, promoting re-polarization of the M1 to M2 phenotype. This opens a new avenue for the study of kaempferol-mediated nanomedicines in bionanoparticle-based atherosclerosis therapy (Zhao et al., 2023).

In fact, reducing lipoproteins to an extremely low level in the early stages of life could theoretically eliminate cardiovascular disease, but this has not been widely used for reasons such as low adherence (Chen et al., 2022). Safety issues have not been fully assessed in many pharmaceutical studies and clinical applications which remains highly challenging. New anti-inflammatory therapies could be developed for therapeutic use to reduce the risk of cardiovascular disease, alleviate associated symptoms and reduce patient suffering.

### 2.4 Inflammation in liver diseases

Chronic liver disease is a highly morbid and ongoing process inextricably linked to liver inflammation and oxidative stress, including various diseases such as chronic viral infections and autoimmune hepatitis, which lead to cirrhosis, fibrosis and liver failure (Hashim et al., 2022; Zhang C. Y. et al., 2023; Foghis et al., 2023). The effectiveness and safety of NPs have been favored by many scientists in recent years due to the range of serious adverse reactions caused by drugs. Several NPs systems such as gold, silver, selenium, and nanomicelles have been extensively studied for their various manifestations in chronic liver disease, but there are still many limitations and challenges that hinder clinical use (Casals et al., 2021; Ezhilarasan, 2021; Hashim et al., 2022).

Hepatitis is the most common cause of liver disease and patients are usually treated with antiviral therapy, combination therapy or alone, as well as liver transplantation in severe cases (Hashim et al., 2022). Recently, the rapid transmission and control of certain epidemics have demonstrated the importance and necessity of targeted vaccines. AbdelAllah NH et al. evaluated the effect of alginate-coated chitosan NPs with average size of 654 nm as adjuvants for hepatitis A vaccine in mice, which significantly improved immunogenicity by increasing seroconversion (100%), hepatitis A antibody levels, and splenocyte proliferation. This adjuvant has advantages in IFN-γ and IL-10 development, suggesting that chitosan alone could replace alum as an adjuvant for hepatitis A virus, providing a new direction for hepatitis A vaccine improvement (AbdelAllah et al., 2020). In addition, the various natural substances present in many medicinal plants are applied in all kinds of fields relevant to the management of liver diseases (Foghis et al., 2023). Mulberry pigment-loaded chitosan NPs were developed to improve the antioxidant system and prevent apoptosis and inflammation in mice, leading to the inhibition of arsenic-induced liver injury. This research breaks through limitations such as the insolubility of mulberry pigments that are unfavorable for therapeutic applications, making mulberry pigment-loaded chitosan NPs a better hepatoprotective agent against arsenic toxicity compared with free mulberry pigments (Mondal et al., 2022).

However, it is impossible to achieve complete hepatitis prevention and how to approach the early diagnosis is also very important as an entry point. The sandwich immunosensing platform based on Au NP-Thionine is capable of capturing higher signal generating sites and has a lower detection limit in serum samples than conventional methods such as enzyme-linked immunosorbent assay for the detection of hepatitis B virus surface antigen, providing a reliable sensing mechanism for hepatitis B diagnosis with certain guiding implications (Hallaj et al., 2022). In addition to antigen testing, convenient, rapid and sensitive diagnostic strategies for hepatitis B virus DNA testing are also significant. A recent study developed a novel sensitive fluorescent biosensor for hepatitis B virus detection based on the CRISPR-Cas12a enzyme and metal nanoclusters as a sensing system for luminescent nanoprobes. The results show that copper nanoclusters probes, averaging about 2.3 nm in diameter, can be successfully used for fluorescence detection of DNA targets with high sensitivity and selectivity, requiring little equipment or reaction conditions and can be completed within 25 min (Tao et al., 2022).

Chronic hepatitis virus infection often progresses to liver fibrosis, which can eventually lead to cirrhosis and liver failure (Wang et al., 2016; Lawitz et al., 2022). Sung YC et al. demonstrated that co-administration of sorafenib and MEK inhibitors via CXCR4-targeted NPs of 140 nm mean diameter blocked ERK activation in activated hematopoietic stem cells and had antifibrotic effects in CCL_4_-induced mouse models, noting the inhibitor’s potential role in controlling fibrosis and preventing malignant disease progression (Sung et al., 2018). Furthermore, a recent trial showed that retinoid-conjugated lipid NPs, BMS-986263, which contained HSP47 siRNA, improved liver fibrosis in patients with HCV-sustained virologic response, and was well tolerated during week 36, warranting further evaluation in patients with active fibrosis (Lawitz et al., 2022).

Taken together, the mechanism of hepatitis becomes more complex, which is associated with a variety of risk factors and underlying conditions including viral infections, bacterial infections and so on. Traditional anti-hepatitis drugs have poor bioavailability due to factors such as resistance, limiting their widespread application in the treatment of liver injury. Antioxidants and anti-inflammatory agents from natural products are a new effective therapy. In relevant clinical models, various nano-agents are used to inhibit liver fibrosis, reduce connective tissue proliferation, and prevent the development of cirrhosis, making them effective strategies for treating liver diseases.

### 2.5 Inflammation in gastrointestinal diseases

Epithelial cells are the initial cells in the skin and oral mucosa that encounter foreign particles by secreting various relevant factors to initiate and coordinate the immune response (Coutinho Almeida-da-Silva et al., 2023). Inflammatory regions of the gastrointestinal tract (GI) require maximum local drug exposure, and pharmacological treatment of GI diseases (e.g. inflammatory bowel disease, IBD) deserves further investigation in terms of targeted delivery to avoid the delivery of non-specific drugs to healthy tissues and limit systemic absorption (Bertoni et al., 2020). Engineered nanomaterials are present at every turn of life and can interact with the intestinal epithelial tissue to induce inflammation through pathways such as inflammatory vesicle activation, which leads to pathology (Coutinho Almeida-da-Silva et al., 2023).

IBD, primarily affects the ileum and colon, and is a chronic, non-specific inflammatory GI disease that requires rigorous testing and long-term treatment (Bertoni et al., 2020; Li et al., 2023b; Coutinho Almeida-da-Silva et al., 2023). Natural active small molecules are widely used in the prevention and relief of IBD, while NPs can effectively encapsulate multi-purpose natural active small molecules, overcoming the limitations of their clinical application such as instability and low bioavailability, enhancing the efficiency of oral drug delivery (Zu et al., 2021a). Zu M et al. developed 140 nm natural exosome-like nanotherapeutic formulations derived from tea leaves that inhibit pro-inflammatory cytokine expression, reduce oxidative stress, and promote the secretion of anti-inflammatory IL-10 from macrophages. These nanotherapeutics help maintain intestinal flora homeostasis and can be targeted for the prevention and treatment of IBD through the oral route (Zu et al., 2021b).

In recent years, there has been widespread interest in the diagnosis and treatment of IBD, which occurs throughout the world, with the incidence increasing every year and getting younger (Fan et al., 2021; Yang et al., 2022). The researchers have explored the value of aggregation-induced emission nanoprobe BPN-BBTD NPs in the second near-infrared (NIR-II) fluorescence imaging for IBD diagnosis and surgery using an IBD mouse model. With the aid of NIR-II fluorescence wide-field microscopy, the distribution of NPs can be detected directly at the tissue level. This method can accurately track inflammatory lesions, monitor the severity of inflammation, and detect the response to pharmacological intervention, indicating the potential and benefits of aggregation-induced emission NPs-assisted NIR-II fluorescence imaging in the diagnosis and surgery of IBD in the future (Fan et al., 2021). Computed tomography imaging is an important diagnostic technique used in various fields of medicine. IBD imaging poses certain challenges due to the low X-ray absorption in soft tissues and the lack of specificity in contrast agents. Therefore, bismuth-based compounds are considered to be the best candidates for human GI computed tomography imaging due to their high X-ray absorption properties. The authors designed polyethylenimine-coated bismuth oxide chloride nanosheets with a flake shape measuring 160 ± 56 nm × 18 ± 5 nm. With exceptional stability and safety, it effectively enhanced computed tomography effects as an *in vitro*/*vivo* X-ray imaging contrast agent. Region-selective computed tomography imaging of the gastrointestinal tract using NPs coated with differentially charged polymers through pH-controlled aggregation in the stomach lays the foundation for advancements in GI clinical diagnosis (Zelepukin et al., 2022).

IBD cannot be completely cured at present because it is highly susceptible to various complications and other diseases, which seriously affect the quality of life of patients and lead to a high mortality rate (Xu et al., 2022; Li et al., 2023a). Au NP-embedded ceria nanoenzyme of 22 nm with ROS scavenging activities has been developed for the treatment of ulcerative colitis. It effectively alleviating colonic damage in colitis mice by reducing inflammatory cell infiltration and production of pro-inflammatory cytokines IL-1β, IL-6, and TNF-ɑ in colitis mice (Li et al., 2023a). This study offers a promising antioxidant nanotherapy for the treatment of colitis. Shi C et al. coupled polyethyleneimine with antioxidant diselenium-based bridged mesoporous organosilica NPs, which not only reduced ROS-mediated pro-inflammatory responses but also blocked the toll-like receptors 9-myeloid differentiation factor 88- nuclear factor kappa-B signaling pathway induced by pro-inflammatory cell-free DNA, reducing dose frequency through preferential accumulation while preventing tissue damage (Shi et al., 2022).

NPs can accumulate specifically at the disease site, making disease detection and diagnosis more efficient and sensitive. Due to the specificity of cells in the inflamed tissues of IBD, recent research has focused on oral drug delivery, with the development of oral NP formulations to deliver drugs to inflammatory sites with precise targeting ( Xiao et al., 2022 ). NPs can enhance the therapeutic effect by anti-ROS or inhibiting the release of pro-inflammatory factors, for example. Emerging nanotechnologies have enormous potential to provide new insights into diagnostic and therapeutic developments for diseases.

### 2.6 Inflammation in kidney diseases

The development of inflammation is the main response to kidney injury associated with uremia, causing renal fibrosis and ultimately premature death from end-stage renal disease or chronic kidney disease (CKD) (Ebert et al., 2020; Puthumana et al., 2021). In addition, it has been suggested that diabetic nephropathy (DN) is also a major cause of end-stage renal disease (Matoba et al., 2019). The combination of NPs and animal models has made it easier to conduct studies and obtain results, but there are still limitations due to the lack of specific protocols for risk assessment (Martínez-Esquivias et al., 2021). In this background, nanomedicine continues to offer new ideas in clinical practice and several nanomaterial-based approaches have been applied in kidney treatment and regeneration, hopefully improving the efficiency of kidney disease treatment and reducing patient suffering (Eftekhari et al., 2021).

Despite the widespread use of NPs in medicine, they are a potentially dangerous chemical that can penetrate animals’ organs and cause functional impairment. Related studies suggest that treatment with melanin, quercetin, and alpha-lipoic acid prevents most of the kidney-damaging effects of Au NPs (Alshammari et al., 2023). Abdelhalim et al. showed that serum renal function biomarkers were significantly increased in rats treated with Au NPs and demonstrated that vitamin E and alpha lipoic acid had beneficial protective effects against Au NP-induced nephrotoxicity, lipid peroxidation, and inflammatory kidney injury (Abdelhalim et al., 2020). Cisplatin is an effective chemotherapy drug. However, repeated administration can lead to nephrotoxicity. Encapsulation of kidney-secreted survival protein renalase agonist peptide (RP81) in 400 nm poly (lactic-co-glycolic acid) NPs functionalized with polyethylene glycol enables specific delivery to the renal proximal tubule. Renal-targeted delivery of the RNLS agonist RP81-mesoscale NP attenuates cisplatin-induced renal tubular injury, oxidative stress, and inflammation, inhibits inflammatory macrophages and myofibroblasts, and has the potential to be an effective therapeutic agent for the prevention of CKD in patients with repeated cisplatin (Guo X. et al., 2022).

It is essential to optimize the diagnosis of kidney disease in the context of kidney injury because the common functional tests such as serum urea and creatinine assays, are not conducive to distinguishing early stages of kidney injury (Bashandy et al., 2022)that are often accompanied by inflammation. A stress-free electrochemical sensor device equipped with the Internet of Things is capable of non-invasively monitoring creatinine in saliva through the irreversible binding of Cu^2+^ ions to the C═N functional group of creatinine. Used for preventative diagnosis and clinical evaluation of CKD, it proves the feasibility of detecting salivary creatinine through a catalytic mechanism to assess the extent of kidney disease (Kalasin et al., 2020). In addition, the rapid distribution and long retention time of bioimaging probes in the kidney are of great importance in the accurate diagnosis of diseases. A Bilateral NIR-II sensor based on renal-targeted peptides and ROS response-activated allows long-term renal monitoring and *in vitro* urine analysis, with coupling to kidney-clearable ultrasmall NPs that extend their retention time in kidney, such as gold NPs, quantum dots and others. It can be used to detect renal insufficiency and will be particularly important for optimizing chemotherapy regimens and nephroprotective interventions (Chen et al., 2021). However, further investigation is needed to elucidate its complete metabolic mechanism.

The protective effects and mechanisms of polydatin have not been fully elucidated as a potential antioxidant, anti-inflammatory and nephroprotective agent against early DN. Abd El-Hameed et al. demonstrated that polydatin-loaded chitosan NPs were able to restore the balance between pro- and anti-inflammatory cytokines. These cytokines could inhibit streptozotocin-induced early progressive diabetes mellitus by NF-κB in the kidney to suppress oxidative stress and renal inflammation, and produce antidiabetic effects (Abd El-Hameed, 2020). In addition, subspherical Mn_3_O_4_ NPs of 5.58 ± 2.42 nm moderately functionalized with the biocompatible ligand citric acid may have great potential in CKD treatment. This redox nanomedicine can regulate redox homeostasis, downregulate pro-inflammatory cytokines, and attenuate renal injury with tubular intestinal fibrosis by synchronizing the causal relationship between mitochondrial protection and ROS scavenging (Adhikari et al., 2021). Few published articles have utilized redox modulation approaches to treat chronic diseases, and this field remains to be continually explored.

Lipids and calcium phosphates are the main insoluble substances found in mammals. They are adsorbed by serum proteins to form colloidal particles found in organs and blood (Kuro, 2021). And CKD patients have a higher prevalence of vascular calcification (Silaghi et al., 2020). Colloidal NPs of calprotectin particles formed in the blood can induce cell damage, ectopic calcification, and inflammatory responses, and have been proposed as new therapeutic targets for age-related diseases, including CKD (Kuro, 2021), which opens up new directions for treatment. Redox NPs (RNPs) can accumulate at inflammation sites and scavenge ROS from damaged tissues, showing significant therapeutic effects on various oxidative stress disorders with the potential to become antioxidant nanomedicines (Yoshitomi and Nagasaki, 2022). Comparatively, the potential dangers of NPs cannot be ignored, which requires more attention in research and clinical trials. Interestingly, CKD is also associated with gut ecological dysbiosis. The interventions to restore the gut microbiota to reduce CKD gut ecological dysbiosis, such as plant-based and low-protein diets, have emerged as potential options for its prevention and treatment (Sumida et al., 2021).

### 2.7 Inflammation in neurological diseases

The underlying causes of neurological disorders are complex and varied and may originate from viral infections, degeneration, trauma, tumors and metabolic diseases that affect blood vessels and neurons (Cerqueira et al., 2020; Pathak and Sriram, 2023). Neuroinflammation is a biological response of the neuroimmune system caused by various agents mainly characterized by a complex inflammatory process in the central nervous system (CNS), including Alzheimer’s disease (AD), Parkinson’s disease, multiple sclerosis and so on. Characteristic features are leukocyte invasion of CNS and disruption of blood-brain barrier (BBB) integrity (Cerqueira et al., 2020; Caminade et al., 2022; Moradi and Dashti, 2022; Pathak and Sriram, 2023). Current therapies are limited in their therapeutic benefits by low drug absorption rates and insufficient drug concentrations in affected CNS areas (Moradi and Dashti, 2022).

It has been shown that redox-regulated Mn_3_O_4_ nanozyme with multi-enzyme activity provides effective cytoprotection of human cells in Parkinson’s disease models, and has therapeutic efficacy in preventing ROS-mediated neurological disorders, which could be a potential candidate for the treatment of oxidative stress-induced neurological disorders (Singh et al., 2017). CNS diseases pose a major threat to human health, and BBB’s presence makes targeted drug delivery a huge challenge. Engineered nanomaterials can be used to provide neuroprotective strategies to overcome the challenges to some extent (Alhibshi et al., 2020). The researchers developed naringenin-loaded solid lipid NPs of 210 nm and showed that the NPs effectively enhanced the neuroprotective effects of naringenin by inhibiting autophagy and enhancing mitochondrial membrane potential, which may be a promising option for the prevention of neurological disorders through autophagy inhibition (Conklin et al., 2022; Nouri et al., 2022). There are numerous natural substances found in nature. The neuroprotective potential of luteolin’s multi-targeted action in neurological disorders has been reviewed, and it has been suggested to prevent neurological disorders through dietary luteolin delivery interventions for its application in the neurological field (Chen et al., 2023).

The incorporation of nanocomposites allows a greater number of active sites and functions to exist in the sensor, enabling the detection of low biomarkers’ concentrations when tested in complex matrices (Kamal Eddin and Fen, 2020; Macovei et al., 2023). This advantage cannot be overlooked in neurological disorders that require rapid diagnosis. Dopamine is an extremely important catecholamine neurotransmitter, which is distributed in the CNS, affects the human body in many fields such as cognition, emotion and desire, and is closely related to neurological disorders (Kamal Eddin and Fen, 2020). Chen T W et al. have developed a green and efficient method for synthesizing CeO2 sheets of 70–85 nm average diameter modified with highly conductive copper oxide NPs to prepare a sensitive, selective electrochemical and altered dopamine sensor. Its modified electrode was established for real-time application in the determination of dopamine in different serum and drug samples (Chen et al., 2020). In addition, the proteins β-amyloid (Aβ) and Tau have become central biomarkers of AD, providing precise diagnostic information for the development of AD treatment through quantification. Metal NPs-based optical and electrochemical biosensors, such as Au, Cu^2+^, Eu^3+^ and so on, have been prepared for measuring β-amyloid and Tau proteins and demonstrated in clinical trials for the early diagnosis of AD, thereby promoting the development of primary Alzheimer’s care interventions (Phan et al., 2021).

AD has become a major health problem affecting the lives of older people. Bashir D J et al. demonstrated that magnoflorine-loaded chitosan collagen nanocapsules of 12 ± 2 nm could improve the cognitive deficit in scopolamine-induced AD rat model through down-regulating inflammation-related cytokines such as IL-1β, IL-6, TNF-α, and oxidative stress, as well as up-regulating brain-derived neurotrophin and DCX expression, which is expected to be an ideal drug for treating AD (Bashir et al., 2023). Interestingly, in Phase 2a clinical trial, the authors found that OP-101, a hydroxy dendrimer coupled with N-acetyl cysteine for the treatment of severe COVID-19, attenuated markers of inflammation and nerve damage, and had good tolerance, which showed potential as a nanomedicine for the management of systemic inflammation and nerve damage (Gusdon et al., 2022).

Nanomaterials can be effectively involved in the prevention, detection and treatment of health problems (Ebrahimi et al., 2021). Optimization of analyte detection processes using nanomaterials can effectively facilitate electron transfer during electrochemical processes and improve diagnostic sensitivity. CNS is one of the most important systems in our body. Abnormal hormones can lead to a variety of physical complications or even cause the onset of disability, making it important to consider the diagnosis of different hormones (Negahdary et al., 2022). Nano-engineered molecules could perform a variety of tasks, such as making it easier to cross BBB, targeting specific cells or signaling pathways, acting as vectors to assist gene delivery to support neuroregeneration and cell survival, and showing greater efficiency in the treatment of neurodegenerative diseases (Bashir et al., 2023; Kumar et al., 2023).

### 2.8 Inflammation in cancer

Chronic inflammation is one of the culprits in the development and progression of cancer (Mantovani, 2018; Greten and Grivennikov, 2019; Neurath, 2020). Inflammation promotes all stages of tumorigenesis and may interact to form an inflammatory tumor microenvironment (TME) in which cells can change changing phenotypic and functional characteristics with a high degree of plasticity (Greten and Grivennikov, 2019). Growing evidence suggests that TME is a key determinant of the effectiveness of conventional chemotherapy and immunotherapy. Various signaling pathways have been identified as key regulators of inflammation initiation and resolution (Zhao et al., 2021). Therefore, it is necessary to research inflammation in cancer to help the course of the disease and its treatment.

Epstein-Barr virus (EBV), mainly transmitted through the oral cavity, is a pathogen associated with cancer, multiple sclerosis and RA, causing hundreds of thousands of deaths each year. The development of an EBV vaccine to prevent this infection is an urgent priority. Malhi H et al. demonstrated that several EBV glycoprotein (gH/gL) NP vaccines were able to elicit an effective neutralizing antibody response and pointed out that a 60-mer NP could protect humanized mice against the lethal challenge of EBV, which have higher immunogenicity compared with gH/gL alone, emphasizing that gH/gL vaccine-induced antibodies have great potential in the development of EBV virus vaccines (Malhi et al., 2022). In another study, a multifunctional spore-encapsulated probiotics nanomaterial of 100 nm was innovatively prepared for oral probiotic delivery. The material can effectively inhibit IL-6 and signal transducers and activators of transcription 3 signaling pathway, restore intestinal barrier integrity and maintain mucosal homeostasis, significantly improve microbiome regulation, with good anti-inflammatory effects and certain tumor prevention. It has good anti-inflammatory effects and a certain tumor prevention effect (Song et al., 2021). All these efforts offer some strategies for tumor prevention to assist in the development of nanomaterials and materials-based tumor prevention agents.

Breast cancer continues to afflict women around the world. Determining the regional lymph node status of breast cancer is critical to understanding the course of breast cancer, and the appropriate tracer of sentinel lymph node biopsy plays a key role in diagnosis and axillary staging. Dual tracer-guided techniques are the current standard for detecting sentinel lymph nodes. Zhang L et al. developed an ultrasound-assisted nanocarbon suspension localization technique for early breast cancer patients using dual tracer-guided sentinel lymph node biopsy and proved that the diagnostic performance of ultrasound-assisted carbon NP suspension mapping in early breast cancer patients was not inferior to the dual tracer-guided technique sentinel lymph nodes mapping combined with carbon NP suspension and indocyanine green, which has potential clinical value in patients receiving neoadjuvant chemotherapy (Zhang L. et al., 2022). However, the current practice of sentinel lymph node localization is limited by the inability to visualize lymph nodes in a high contrast and sensitive manner. A non-randomized clinical trial of image-guided sentinel lymph node biopsies of head and neck melanoma using ultra-small core-shell fluorescent silica NPs of 6.4 nm has shown that it is safe to use intraoperatively at nanomolar doses for visual identification of solid lipid NPs in head and neck melanoma patients (Zanoni et al., 2021). The surgeon’s experience in clinical practice is crucial, and this new particle-based technique not only significantly changes routine surgical practice and improves intraoperative safety, but also assists the surgeon in obtaining more consistent clinical results at the time of biopsy.

Conventional cancer treatment strategies have limitations in the clinical setting with inherent physiological barriers such as drug resistance and drug delivery (Ang et al., 2021; Gao et al., 2022). In recent years, nanomaterials have been extensively studied in the diagnosis and treatment of tumors, offering promising strategies and demonstrating good application prospects (Ang et al., 2021; Nguyen and Lai, 2022; Luo et al., 2023). A brain-infiltrating RNA interference-based spherical nucleic acids composed of Au NP cores linked to siRNA oligonucleotides has been developed for glioblastoma therapy (Kumthekar et al., 2021). This is the first human phase 0 clinical study of RNA interference-based spherical nucleic acids in patients with recurrent glioblastoma, and the safety profile will be fully evaluated in future clinical trials to increase patient survival. Zhang H et al. constructed a manganese oxide-based artemisinin co-delivery system, TKD@RBCm-Mn_2_O_3_-ART. The particle size of synthetic hollow mesoporous manganese trioxide NPs was reduced from 6 nm to 1 nm and 4 nm with time in the simulated TME. Co-delivery of Mn^2+^ and artemisinin, as well as modification of homologous erythrocyte membranes and TKD peptides, resulted in prolonged circulation and tumor targeting in the body. It shows an excellent imaging ability in tumors, generates large amounts of ROS, and induces DNA damage, enabling accurate breast cancer diagnosis and low toxicity treatment (Zhang et al., 2021a).

Taken together, these studies hold the promise to improve sentinel lymph node biopsy procedures while potentially reducing the present procedural risks. There is an increasing focus on health issues and continuous attempts to optimize existing treatment options and develop new potential drugs (Zhang et al., 2022b; Xu et al., 2023). However, due to the limitations of the trial population, further studies in other populations are needed before widespread use can be achieved. The widespread use of some tumor models and their combination with traditional or emerging nanomaterial-based models may address some limitations of existing preclinical tumor models and open up new directions for preclinical research efforts (Mapanao et al., 2021). At the same time, it is also important to consider the practicality of exploring other treatments to eliminate immunosuppressive interactions and immune-related side effects, provide cancer cell targeting and prevent tumor recurrence (Lima-Sousa et al., 2021). Above all, to activate relevant immune remodeling of TME and enhance immunotherapy, TME-responsive nanomaterials show great potential in cancer therapy (Fan and Guo, 2023).

## 3 Discussion and conclusion

Early and accurate detection of disease is vital, but the processes involved in diagnosing certain diseases are very time-consuming. Many diseases have identical or closely similar characteristics that cannot be separated from the onset of inflammation, making them difficult to determine. Therefore, diagnostic modalities and differential criteria need to be further refined. In order to overcome the inflammation seen in various diseases, nano-agents have received a lot of attention from researchers in recent years. Encapsulation of drugs in NPs can improve efficacy and reduce the occurrence of side effects, as well as provide good biocompatibility and safety. Moreover, nanocarriers can cross the human barrier by various routes due to their small particle size, thereby improving drug delivery efficiency. Some drugs and natural compounds are limited in application due to their physicochemical properties (e.g. insolubility) and site specificity. For various drug delivery routes, NPs are highly flexible and can be incorporated into various dosage forms such as capsules, tablets, and hydrogels (Bilia et al., 2018).

Immune-related macrophages play an important role in inflammation and body defense. Moreover, chronic inflammation is inextricably linked to macrophage activation (Louiselle et al., 2021; Yuan et al., 2022). Macrophages are polarized into M1 and M2 phenotypes and regulate inflammation mainly through the release of pro/anti-inflammatory cytokines and various chemokines (Lee et al., 2020). In the early stages, M1 macrophages play a major role, producing pro-inflammatory mediators and interacting with Th1 cells. Subsequently, M2 macrophages produce anti-inflammatory factors that promote Th2 responses and adaptive immune systems. The M1-M2 transition can regulate the inflammatory immune environment, ensuring tissue remodeling and wound healing while preventing further damage (Joorabloo and Liu, 2022). Conventional drug therapy is prone to adverse effects, while NP-based nanomedicines achieve pharmacological effects with fewer adverse effects (Cao et al., 2022). Research has identified M2-type exosomal NPs loaded with betamethasone sodium phosphate as a promising drug carrier and anti-inflammatory agent for RA by targeting and reducing inflammation through macrophage repolarization (Li et al., 2022a). In addition, some natural compounds can modulate macrophage polarization progression and have great potential in the treatment of chronic inflammation-related diseases (Wang et al., 2019). However, their specific mechanisms need to be further investigated.

However, despite the advantages of functional materials-based nano-formulations for drug delivery and inflammation treatment, materials may also cause worsening inflammation, mostly associated with increased ROS production (Palmer et al., 2019; Jogpal et al., 2022; Parhiz et al., 2022; Midander et al., 2023). Thus, nanomaterials can be a double-edged sword. NPs can interact with the immune system in many different ways. Studies have shown that engineered nanomaterials may elicit either acute or chronic inflammation with engagement of neutrophils, macrophages and other effector cells. Cho et al. focused on pulmonary responses to nanomaterials and found that CeO_2_ NPs, NiO NPs, ZnO NPs, and CuO NPs induced a unique inflammatory “footprint” both acutely and chronically with different patterns of neutrophil and eosinophil infiltration elicited by different NPs (Cho et al., 2010) The studies conducted by Boukholda et al. provided new insights into the molecular mechanism of Silica NPs-induced oxidative stress and inflammation in the hippocampus, as well as disruption of the cholinergic system and behavioral functions (Boukholda et al., 2021). Another finding indicated that lipid NPs (composed of ionizable cationic lipid, phosphatidylcholine, cholesterol, and polyethylene glycol-lipid) induced inflammation exacerbation in gram negative bacterial inflammation by enhancing inflammatory cytokine responses, IL-6 in serum, and Macrophage Inflammatory Protein 2 in liver significantly, which was shown to be lipid NPs-specific (Parhiz et al., 2022). However, the generalizability of these lipid NPs in other forms of chronic or acute inflammatory and immune contexts needs to be addressed. NPs can also cause a variety of unpredictable toxicities that threaten human health. Silver NPs can induce microglia polarization of inflammatory phenotype, and hinder autophagic flux by inhibiting autophagosome fusion with lysosomes, thus exacerbating neurotoxicity induced by silver NPs (Shang et al., 2022) Currently, only a limited number of nanomaterials have entered clinical trials or are available to treat chronic inflammatory diseases (Huang et al., 2020; Placha and Jampilek, 2021). In addition, there is no unified internationally recognized standard for measuring the treatment and safety of nanomaterials yet. Existing studies have identified only certain materials that may induce inflammation or other toxic effects. The selection of these materials for drug delivery in chronic inflammation should be based on their efficacy and potential limitations as delivery carriers. Therefore, extensive experiments and theories are needed to understand these cytotoxic effects, and more clinical studies are needed to validate them (Xiong et al., 2022). A recent study proposes a boron-capture strategy for bacterial infections and related inflammation. Such reactive magnesium metal borides NPs not only disrupt bacterial membrane structure, but also trap lipopolysaccharides or peptidoglycans released by dead bacteria, preventing infection and excessive inflammation (Meng et al., 2022).

In summary, nanomedicine introduces new preventive, diagnostic and therapeutic drugs that can more efficiently integrate effective molecules and utilize their high flexibility and durability combined with a variety of materials to protect the drug from degradation, provide precise targeting, effective control and specific release, as well as reduce toxicity and improve drug therapeutic efficacy. However, the current therapeutic efficacy is still mainly limited to animal models, and further studies are needed to elucidate the protective effects on humans.
