# Supplementary material for: The applications of functional materials-based nano-formulations in the prevention, diagnosis and treatment of chronic inflammation-related diseases
Source: Front Pharmacol. 2023 Aug 1;14:1222642. doi: 10.3389/fphar.2023.1222642 (PMC10427346; doi:10.3389/fphar.2023.1222642)
Supplement: Supplementary file 1 [file Table2.docx]

Table 2. Scientific research on nanomaterial formulations in inflammation-related diseases

| Disease category | Disease name | Nanomaterials | Drug | Size  (nm) | Action mechanism | | Outcome | Level study | | References |
| --- | --- | --- | --- | --- | --- | --- | --- | --- | --- | --- |
| skin diseases | psoriasis | fluidized-transplasmic nanovesicles of 1% eucalyptus oil brain | Tazarotene | 241.5 ± 5.68 | | direct delivery in skin stratification, deposition | dermoscopic imaging and morphological analysis | ex vivo | (Elmowafy et al., 2019) | |
|  |  | nanocurcumin | Acitretin | 785.50 ± 30.16 | | inhibit crystal growth, prevent crystals from interacting with dissolved drugs, increase water solubility | treatment with psoriasis (PASI ≥ 10), prevention of avelic acid-induced hypercholesterolaemia | clinical trial | (Bilia et al., 2018) | |
|  |  | chitosan hydrogel | Methotrexate | 256.4 ± 2.17 | | reduce TNF-α and IL-6, enhance solubility, better skin deposition | improve local effectiveness in psoriasis management | in vitro/  in vivo | (Asad et al., 2021) | |
|  |  | gold NPs with peptide ligand | γ- glutathione | 2.04 | | repress TNF-α/NF-κB/IL-17A axis in keratinocytes | high biosafety and biocompatibility in treatment | in vitro/  in vivo | (Liu et al., 2023) | |
|  |  | HA-US-His micelles | Shikonin | 130.21 ± 7.55 | | enhance regulation of Th17-related cytokines, matrix metalloproteinases and chemokines | active epidermal targeting delivery system for topical treatment | in vitro/  in vivo | (Jing et al., 2021) | |
|  |  | ZnO NPs | - | 50 | | promote nuclear translocation and cysteine deficiency of p-NFκB p65 | promote inflammation and keratin-forming cell apoptosis | in vitro/  in vivo | (Lai et al., 2021) | |
|  | allergic dermatitis | poly-ε-caprolactone | hydrophobic allergens | < 200 | | increase allergen solubilization, activate allergen-specific T cells | improve the in vitro immunobiological diagnosis | in vitro | (Cortial et al., 2015) | |
|  |  | chitosan/hyaluronate hybrid NPs | Etoricoxib | 267.9 ± 9.4 | | reduce TNF-α and ALP/MPO enzyme activity | enhance skin delivery of lipophilic drugs | in vivo | (Abuelella et al., 2023) | |
|  |  | Co NPs | - | < 100 | | S100A9, IL22, BATF, Th1 and Th2 signaling induction, down regulation of FLG and SPINK5down regulation | co-induced immune responses are independent of exposure form | in vitro/  ex vivo | (Midander et al., 2023) | |
|  | atopic dermatitis | chitosan NPs | hydrocortisone, hydroxytyrosol | < 250 | | - | safe and well-tolerated, with no systemic absorption or toxicity | in vivo | (Siddique et al., 2019) | |
|  |  | L-ascorbic acid-loaded poly-γ-PGA microneedles | epigallocatechin gallate | - | | down regulation of serum IgE and IgG1 levels | transdermal immunomodulators for treatment | in vivo | (Chiu et al., 2021) | |
|  |  | γ-Fe_2_O_3_ NPs | Hederagenin | 10.9 | | reduce mRNA expression of TNF-α, IFN-γ, IL-4, IL-6, IL-17 and TSLP | substitution of non-steroid-based medication for treatment | in vivo | (Lee et al., 2022) | |
|  | acne | electrostatic spinning, gelatine nanofibers | lavender & peppermint essential oils | 430 | | anti-oxidant and anti-bacterial | topical antimicrobial agent after incorporation of gelatin nanofibers | in vitro | (Uhlířová et al., 2023) | |
|  |  | Au-Ag-PDA nanoparticle | - | - | | inhibit sebaceous gland cell proliferation, reduce sebaceous gland size and sebum production | potential clinical treatments for acne | in vitro/  in vivo | (Meng et al., 2020) | |
|  |  | gold nanostars | chitosan, ascorbic acid | from 14.0 ± 4.4 to 54.2 ± 17.9 | | disrupt bacterial cell membranes, inhibit protein processes | addressing antimicrobial resistance in treatment | in vitro | (Huynh et al., 2021) | |
| rheumatoid arthritis | | plant virus NPs | tomato bushy stunt virus | 30 | | depletion of TNF-α, IL1β, IL17 and IFN-γ in serum, lymph nodes and joints | reducing RA severity | in vivo | (Zampieri et al., 2020) | |
|  |  | polymeric α-ketoglutaric acid NPs | Methotrexate | 191.3 ± 16.2 | | down regulation of Th17 bc2-specific antigen responses, enhancement of Th2 type | simultaneous, short-term, low-dose combination reverses RA symptoms | in vitro/  in vivo | (Mangal et al., 2022) | |
|  |  | polyethyleneimine coated gold NPs | mucic acid | 80 | | reduce serum production of anti-bovine type II collagen antibodies | reduce pro-inflammatory component, potential anti-arthritic drug | in vivo | (Cui et al., 2022) | |
|  |  | nanoemulsion | Methotrexate | 87.89 ± 2.86 | | subcutaneous delivery of drug-laden nanomolecules for uptake into the lymphatic fluid | reduce systemic toxicity and improves therapeutic efficacy | in vitro/  in vivo | (Suresh et al., 2022) | |
|  |  | Prussian blue NPs | sinomenine hydrochloride | 110.2 ± 12.35 | | inhibit TNF-α and G-CSF secretion, scavenges ROS | an excellent vehicle for the controlled release and targeted accumulation | in vitro/  in vivo | (Lin et al., 2022) | |
| systemic lupus erythematosus | | dextran NPs | mycophenolic acid | 100 | | promote local m2-like macrophage polarization, down regulation of surface CD80 and CD40, reduce TNF-α production | good long-term safety profile with potential for clinical application | in vitro/  in vivo | (Jiang et al., 2022) | |
|  |  | ICOS/CD40L bispecific NPs | rapamycin | 108 | | reduce IL-21 and IgG levels, inhibits Tfh and B cell proliferation, restore Treg homeostasis | offering new combined strategies for SLE treatment | in vitro/  in vivo | (Zhang et al., 2022a) | |
|  |  | PD-1/TIGIT dual-activating cell-membrane | dexamethasone | 114 | | co-inhibitory receptors PD-1 and TIGIT specifically bind pathogenic CD1 T cells, create effector T cell/Treg balance | tumor membrane NPs offer advantages in SLE treatment | in vitro/  in vivo | (Guo et al., 2022a) | |
|  |  | red blood cell membrane-coated poly lactic-co-glycolic acid NPs | cyclosporin A | 95 | | reduce macrophage internalization, decrease TNF-α and IL-6 levels | positive biosafety to improve treatment outcomes | in vitro/  in vivo | (Hao et al., 2022) | |
| cardiovascular diseases | atherosclerosis | hybrid metal oxide-peptide amphiphile micelles | CREKA peptides | 20 - 30 | | clot-specific enhancement improves binding, twofold enhancement of MR signals | non-invasive imaging method for the specific detection of atherosclerotic thrombosis | in vitro | (Poon et al., 2018) | |
|  |  | graphene oxide quantum dots nanomaterial | atorvastatin | 85 | | inhibit oxygenated LDL-induced foam cell formation, reduce TNF-α, IL-1β, IL-6 and MCP-1 | stabilize atherosclerotic plaques, reduce the risk of myocardial damage | in vitro/  in vivo | (You et al., 2022) | |
|  |  | ceria nanocomposites | zoledronic acid | 10 | | reduce NF-κB P65 protein phosphorylation, down regulation of TNF-α and MMP-9 | ROS scavenging, anti-inflammatory and drug transport capabilities | in vitro/  in vivo | (Fu et al., 2022) | |
|  |  | silica NPs | - | 12.5 / 88 | | endoplasmic reticulum stress-mediated upregulation of CD36 expression | macrophage polarization towards M1 phenotype exacerbates | in vivo | (Stachyra et al., 2022) | |
|  |  | composite mesoporous silica NPs | SRT1720 | 61.4 ± 7.9 | | TC reduction and anti-CD36 targeting of RAW264.7 cells | atherosclerosis lesion tracking, improve therapeutic effect | in vitro/  in vivo | (Wang et al., 2021a) | |
|  | myocardial infarction | graphene oxide nano-formulations | silk protein | - | | reduce mRNA expression and protein levels of pro-inflammatory cytokines without disturbing anti-inflammatory factors | potential applications for regenerative therapeutic cell delivery vesicles | in vitro | (Yuan et al., 2020) | |
|  |  | mesoporous silica NPs | miR-21-5p | - | | inhibit TLR/NF-κB signaling pathway, targets SPRY1 and activate VEGF-induced ERK-MAPK signaling to promote angiogenesis | reduce inflammatory response, modifies tissue remodeling, prevent tissue fibrosis, improve repair | in vitro/  in vivo | (Li et al., 2021) | |
|  |  | gold NPs | *euphorbia fischeriana* | 10 - 52 | | reduce serum cardiac troponins T and I, plasma TBARS and LOOH, reduce TNF-α, IL-6, IL-1β and NF-κB expression | anti-myocardial infarction protection, antioxidant and anti-inflammatory properties | in vivo | (Zhang et al., 2020) | |
|  |  | mesoporous silica NPs | notoginsenoside R1 | 83 | | reduce IL-1β, TNF-α, IL-6 and MCP-1 levels, increase IL-11, VEGF, bFGF, SCF and SDF-1 levels, activate AKT, ERK and YAP signaling pathways | improve NgR1 targeting, modifies inflammation and promote angiogenesis | in vitro/  in vivo | (Li et al., 2022a) | |
| liver diseases | alcoholic liver disease | poly D, L-lactide-co-glycolic acid NPs | tannic acid, vitamin E | 127.5 ± 1.6 | | inhibit EGFR-AKT-STAT3 pathway, decrease expression levels of TNF-α, IL-1β, IL-6, TGF-β, iNOS and COX-2 | liver protection for ALS prevention | in vitro/  in vivo | (Nag et al., 2020) | |
|  |  | exosome-like NPs | *Lactobacillus rhamnosus* GG | 75 | | increase Reg3γ, Reg3β and tight junction protein expression through the AhR-Nrf2 signaling pathway | reduce bacterial translocation endotoxemia, prevent fatty liver and damage | in vitro/  in vivo | (Gu et al., 2021) | |
|  | non-alcoholic fatty liver disease | mPEG-PLGA polymer NPs | rapamycin | 132.6 ± 13.5 | | reduce REBP-1c-dependent *de novo* lipogenesis, promote PPARα-mediated fatty acid oxidation | potential therapeutic strategies and novel modes of administration | in vitro/  in vivo | (Zhao et al., 2020) | |
|  |  | silver NPs | - | 25.96 ± 2.28 | | increase mRNA expression of Srebp-1C, Fasn, Acc1 and Scd1, elevate levels of TNF-α, IL-6 and IL-1β, reduce DNA methylation and hydroxymethylation levels | exacerbate hepatic steatosis, liver inflammation, oxidative stress and epigenetic changes | in vivo | (Wen et al., 2022) | |
|  |  | amorphous selenium nanodots | - | - | | reduce ICAM-1, VCAM-1, IL-1 and IL-6, activate VEGF receptor 1 to inhibit phosphorylation of the JNK/ p38 MAPK pathway | reduce oxidative stress and inflammatory response, improve structure and function of liver | in vivo | (Zhu et al., 2022) | |
| gastrointestinal diseases | colitis | bismuth oxychloride nanosheets | - | 160 ± 56 × 18 ± 5 | | - | high stability and safety with regioselective gastrointestinal X-ray imaging | in vitro/  in vivo | (Zelepukin et al., 2022) | |
|  |  | chitosan/alginate-PLGA-NPs | resveratrol | 255.9 ± 12.0 | | reduce MPO activity, scavenge free radicals | targeting and aggregation properties with good biocompatibility for oral treatment | in vitro/  in vivo | (Jin et al., 2021b) | |
|  |  | macrophage-biomimetic nanomedicine | rosiglitazone | 135.2 | | decrease MPO, CD86, iNOS, IL-1β, TNF-α and IL-6, increase CD206, Arg-1 and IL-10 | polarize macrophages to M2, modulate the inflammatory environment for synergistic therapy | in vitro/  in vivo | (Sun et al., 2020) | |
|  |  | graphene oxide nanomaterials | - | 200 - 300 | | increase IL-17 and IFN-γ, induce MMP loss and ROS production to activate the AMPK/p53 pathway leading to apoptosis | activate ROS/AMPK/p53 signaling pathway to mediate apoptosis, thereby exacerbating colitis | in vitro/  in vivo | (Liu et al., 2021) | |
|  | Crohn's disease | mesoporous organosilica NPs | poly-ethylenimine | 50 | | block cfDNA-induced TLR9-MyD88-NFκB signaling, reduce ROS-mediated pro-inflammatory immune responses | new cfDNA and ROS removal methods for treatment | in vitro/  in vivo | (Shi et al., 2022) | |
|  |  | nanostructured lipid carriers | rifabutin | 111 ± 3-151 ± 34 | | - | good permeability and safety for oral treatment | in vitro | (Rouco et al., 2020) | |
|  |  | titanium dioxide NPs | - | - | | restore cytotoxic CD8^+^ T cells, promote pro-inflammatory macrophage phenotype | adverse effects due to elimination of protection from PTPN22 locus variation | in vivo | (Schwarzfischer et al., 2022) | |
|  | bacterial infection gastritis | zinc-based zeolitic-imidazolate framework | - | 190 | | increase bacterial cell membrane permeability, release Zn, inhibit urease activity, down-regulate NF-κB p8 expression, up-regulate mucosal repair protein expression | metal-organic framework and hydrogen therapy combine for the first time in *H. pylori* treatment | in vitro/  in vivo | (Zhang et al., 2022d) | |
|  |  | mannose conjugated chitosan NPs | poly malic acid | 290 ± 7 | | electrostatic interactions to destroy cell walls, specific adhesion by binding of mannose and FimH proteins, conformational changes by binding of ligands to active site residues | alternative therapies for antibiotic-resistant gastric pathogens | in vitro | (Arif et al., 2022) | |
|  |  | chitosan NPs | *Aloe vera* | 36.54 | | interaction with O 17 and O 23 via 4HI0 protein, H-atom gain/loss and non-covalent molecular interactions of ligands with receptors | anti-*H. pylori* effect with chlorogenic acid and catechol as main components | in vitro | (Yahya et al., 2022) | |
|  |  | Fe_3_O_4_ superparamagnetic NPs | - | - | | capture and scavenging of Fe^3+^, activate re-illumination of quenched CDs | accurate, sensitive and immediate antibody-dependent detection of *H. pylori* | in vitro | (Wang et al., 2021b) | |
| kidney diseases | nephritis | mesoscale nanoparticle | RP81 | 401.9 ± 19.2 | | induce the mitochondrial genes Cytb, Nd1, Nd3, Nd4L and Nd5, reduce IFNγ, IL-1β, IL-6 and TNFα, RIPK1 expression, decrease ERK and JNK activation | protect against cell death, reduce tubular damage, oxidative stress and inflammation to prevent cisplatin-induced CKD | in vitro/  in vivo | (Guo et al., 2022b) | |
|  |  | polydopamine NPs | necrostatin-1 | 142 | | inhibit RIPK1 kinase activity, reduce RIPK1 production of neutrophil extracellular traps. | good safety profile, relieving inflammation, dual-mode imaging guided targeted therapy | in vitro/  in vivo | (Li et al., 2022b) | |
|  |  | glycol chitosan nanomicelles | tacrolimus | 370 ± 22 | | down regulation of TGF-β1 / JNK-p38MAPK signaling pathway, short interfering RNA inhibits MAPK1 gene and protein expression, interferes with NF-κB signaling | regulation of TGF-β1 / MAPK / NF-κB monoculture pathway to prevent inflammation, fibrosis and apoptosis | in vitro/  in vivo | (Kim et al., 2021) | |
|  | diabetic nephropathy | chitosan NPs | polydatin | - | | decrease BG, HbA1c, urea, UA, SCr, IL-6, IL-18 and TNF-α levels, increase IL-10 levels, inhibit AGEs formation, decrease NF-κB and COX-2 protein expression | down regulation of NF-κB and COX-2 to improve the inflammatory response to attenuate early DN | in vivo | (Abd El-Hameed, 2020) | |
|  |  | chitosan selenium NPs | metformin alone | - | | reduce TNF-α, IL-6 and IL-1β, inhibit MAPK and NF-κB pathway activation, inhibit TGFβ2, Akr1b1, desmin, nestin and vimentin pathways signaling | inhibit oxidative stress and alleviate renal cell fibrosis with potential combination therapy | in vivo | (Khater et al., 2021) | |
|  |  | gold NPs | - | 51.8 ± 0.7 | | reduce expression of fibronectin, TGF-β1, MDA, VEGF and mRNA levels of TNF-α | attenuate albuminuria by inhibiting podocyte damage, DN prevention and treatment | in vivo | (Alomari et al., 2020) | |
|  |  | polymeric NPs | insulin | - | | up-regulation of SOD, GSH and T-AOC levels, reduction of MDA, TNF-α, IL-1, IL-6, IL-8 and hs-CRP levels | self-regulating glucose response intelligence system for reducing the incidence of DN | in vitro/  in vivo | (Ma et al., 2022) | |
|  |  | gold NPs | pomegranate peel extract | 20 | | regulate MAPK/NF-κB/STAT3/cytokine axis, reduce LPO and iROS production, attenuate NOX4/p-47^phox^ activation, PI3K/AKT-directed Nrf2 activation | regulate inflammation and fibrous tissue accumulation, prevent oxidative stress and its adverse effects | in vivo | (Manna et al., 2019) | |
| neurological diseases | Alzheimer's disease | chitosan collagen nanocapsules | magnoflorine | 12 ± 2 | | reduce acetylcholinesterase activity, increase BDNF, SOD and CAT levels, decrease IL-1β, IL-6, TNF-α and MDA levels | reduce acetylcholine, memory enhancement, anti-amnesic effect for AD treatment | in vitro/  in vivo | (Bashir et al., 2022; Bashir et al., 2023) | |
|  |  | selenium nanoparticle | resveratrols | 60 - 90 | | Aβ clearance, PI3K activation, down-regulation of STAT3, IL-1β and miRNA-134 levels, up-regulation of SIRT1 expression | improve neurocognitive function and modulates signaling pathways through anti-inflammatory effects | in vivo | (Abozaid et al., 2022) | |
|  |  | mica nanoparticle | STB-MP | L: 450, H: 15 | | reduce TNFα, IL-1α, IL-1β and IFITM3 expression, inhibit mTOR-activated autophagy to reduce Aβ formation | screening platform for the assessment of novel AD treatments | in vitro | (Kim et al., 2023) | |
|  | Parkinson's disease | PEGylated 2D-nanomaterials | - | - | | restore tyrosine hydroxylase expression, shielding PIP2 lipids to inhibit IP3 second messenger signaling | selective modulation of membrane lipid dysregulation, non-invasive treatment of PD | in vitro/  in vivo | (Huang et al., 2022) | |
|  |  | ZnO nanorods | - | D: 159.3 ± 17.9, L: 1.1 ± 0.15 μm | | increase mRNA levels of gap43 and tuba1b, elevate syn2a expression | activate regenerative mechanisms, affect synaptogenesis, toxicity and its potential risk for PD development | in vitro/  in vivo | (Jin et al., 2021a) | |
|  |  | nanoscale coordination polymers | curcumin | 10 | | increase DA, DOPAC and HVA levels, decrease ROS and MDA levels | prolong circulation, crosse BBB, scavenge ROS, alleviate inflammation and mitochondrial dysfunction | in vitro/  in vivo | (Cheng et al., 2022) | |
|  | multiple sclerosis | solid lipid NPs | dimethyl fumarate | 315 | | regulate Foxp3+ cells, reduce IFN-γ, TNF-α and IL-17 levels, activate Nrf2 | induce lung Treg cells to suppress local and systemic inflammation in CNS | in vivo | (Pinto et al., 2022) | |
|  |  | high-density lipoprotein-mimicking peptide-phospholipid scaffold | curcumin | 15 ± 2.3 | | reduce ICAM-1 and MAC-1 expression, inhibit NF-κB activation | target regulation of monocytes, new strategies for MS diagnosis and treatment | in vitro/  in vivo | (Lu et al., 2020) | |
|  |  | graphene oxide Nanosheets | - | 100 - 400 | | disrupt HC activation, block inflammation-induced calcium dynamics, down-regulate hemichannel opening and aberrant calcium signaling | specifically target HC permeability in reactive cells and reduce astrocyte responsiveness to inflammation | in vitro/  in vivo | (Di Mauro et al., 2023) | |
| cancer | breast cancer | carbon nanoparticle suspension | - | 150 | | - | ultrasound-assisted mapping of carbon nanoparticle suspensions provides good diagnostic results | clinical trial | (Zhang et al., 2022c) | |
|  |  | PLGA NPs | palmitic acid | 157.8 ± 9.5 | | induce Apaf-1 expression, inhibit Birc5, CD163, Mdr1 expression, promote macrophage reversal to M1 | promote apoptosis through cystein-3 non-dependent pathway | in vitro/  in vivo | (He et al., 2023) | |
|  |  | chitosan NPs | doxorubicin | 154.1 ± 4.8 | | block NF-κB signaling pathway, promote depletion of MDSCs | inhibit pre-metastatic ecotone formation, alleviate inflammation and immune suppression microenvironment | in vitro/  in vivo | (Lu et al., 2022) | |
|  |  | iron oxide NPs | poly amidoamine | 10 ± 4 | | increase Bax expression, decrease Bcl-2 expression, reduce MC4L2 cell proliferation | increase apoptosis, reduce tumor angiogenesis to reduce tumor blood supply | in vitro/  in vivo | (Salimi et al., 2020) | |
|  | lung cancer | carbon black NPs | - | - | | increase IL-6 and MIP-1β, activate TNF-α signaling via NF-κB and EMT pathways | circulating inflammation affects the cell cycle to increase risk | ex vivo | (Zhang et al., 2022b) | |
|  |  | schizophyllan NPs | CpG ODN 1826 | 100 | | increase IFN-γ and IL-1β positivity, elevated levels of Th1 inflammatory cytokines and ROS | increase TLR-9 agonist-mediated immunostimulatory potential, improve bioavailability | in vitro | (Aldawsari et al., 2021) | |
|  |  | cisplatin NPs | vitamin-D_3_ | 68 | | reduce NF-κB, IL-1β, IL-10, Akt, Bcl-2 and TGF-β, Mdr1 levels | new therapeutic approaches in combination | in vivo | (Radwan et al., 2021) | |

**Abbreviations:** PASI, psoriasis area and severity index; TNF-α, tumor necrosis factor-alpha; IL, interleukin; NPs, nanoparticles; NF-κB, nuclear factor-κB; Th, T helper cell; ALP, alkaline phosphatase; MPO, myeloperoxidase; BATF, basic leucine zipper ATF-like transcription factor; FLG, filaggrin; SPINK5, serine peptidase inhibitor kazal type 5; Ig, immunoglobulin; IFN-γ, interferon γ; TSLP, thymic stromal lymphopoietin; RA, rheumatoid arthritis; ROS, reactive oxygen species; G-CSF, granulocyte colony-stimulating factor; CD, cluster of differentiation; ICOS, inducible T-cell costimulator; Tfh, Follicular helper T cell; SLE, systemic lupus erythematosus; PD-1, programmed cell death protein; TIGIT, T cell immunoreceptor with Ig and ITIM domains; LDL, very low density lipoprotein; MCP-1, monocyte chemoattractant protein 1; MMP, matrix metallopeptidase; TC, total cholesterol; TLR, toll-like receptor; SPRY1, sprouty homolog 1; VEGF, vascular endothelial growth factor; ERK, extracellular regulated protein kinases; MAPK, mitogen-activated protein kinase; TBARS, thiobarbituric acid reactive substances; LOOH, lipid hydroperoxides; bFGF, fibroblast growth factor 2; SCF, stem cell factors; SDF-1, stromal cell-derived factor 1; AKT, serine/threonine kinase; YAP, yes-associated protein; NgR1, nogo receptor 1; EGFR, epidermal growth factor receptor; STAT3, signal transducers and activators of transcription 3; TGF, transforming growth factor; iNOS, inducible nitric oxide synthase; COX-2, cyclooxygenase-2; ALS, amyotrophic lateral sclerosis; Reg, regenerating gene; AhR, arylhydrocarbon receptor; Nrf2, nuclear factor E2-related factor 2; mPEG, methoxy polyethylene glycol; PLGA, poly(lactic-co-glycolic acid); REBP-1c, lipogenic genes; PPARα, lipolytic genes; Srebp-1C, transcription factor and lipid homeostasis; Fasn, fatty acid synthase; Acc1, acetyl coenzyme A carboxylase 1; Scd1, stearoyl-CoA desaturase-1; ICAM-1, intercellular cell adhesion molecule 1; VCAM-1, vascular cell adhesion molecule 1; JNK, c-Jun N-terminal kinase; Arg-1, arginase 1; AMPK, adenosine 5’-monophosphate-activated protein kinase; cfDNA, cell free DNA; Myd88, myeloid differentiation primary response protein 88; *H. pylori*, *Helicobacter pylori*; Cytb, cytochrome b gene; Nd, nicotinamide adenine dinucleotide dehydrogenase; RIPK1, receptor-interacting protein kinase 1; BG, blood glucose; HbA1c, blood glycosylated hemoglobin; UA, uric acid; SCr, serum creatinine; AGEs, advanced glycation-end products; DN, diabetic nephropathy; Akr1b1, aldose reductase; MDA, malondialdehyde; SOD, superoxide dismutase; GSH, glutathione; T-AOC, total antioxidative capacity; hs-CRP, high-sensitivity C-reactive protein; LPO, lipid peroxidation; iROS, intracellular ROS; NOX4, NADPH Oxidase 4; PI3K, Phosphatidylinositol-3-kinase; BDNF, brain-derived neurotrophic factor; CAT, catalase; AD, Alzheimer's disease; SIRT1, silent information regulator 1; IFITM3, interferon-induced transmembrane protein 3; mTOR, mammalian target of rapamycin; PIP2, phosphatidylinositol-4,5-bisphosphate; IP3, inositol 1,4,5-trisphosphate; PD, Parkinson's disease; gap43, growth associated protein 43; tuba1b, tubulin alpha 1b; syn2a, synapsin IIa; DOPAC, 3,4-dihydroxyphenylacetic acid; HVA, homovanillic acid; BBB, blood brain barrier; Foxp3, forkhead box protein P3; CNS, central nervous system; MAC-1, macrophage antigen 1; MS, multiple sclerosis; HC, hemichannel; Birc5, Baculoviral IAP repeat containing 5; Mdr1, Multidrug resistance gene 1; MDSCs, myeloid-derived suppressor cells; Bax, BCL2-Associated X; Bcl-2, B-cell lymphoma-2; MIP-1β, macrophage inflammatory protein-1 beta; EMT, Epithelial–mesenchymal transition.

**References**

Abd El-Hameed, A.M. (2020). Polydatin-loaded chitosan nanoparticles ameliorates early diabetic nephropathy by attenuating oxidative stress and inflammatory responses in streptozotocin-induced diabetic rat. *Journal of Diabetes & Metabolic Disorders* 19**,** 1599-1607.

Abozaid, O.A.R., Sallam, M.W., El-Sonbaty, S., Aziza, S., Emad, B., and Ahmed, E.S.A. (2022). Resveratrol-Selenium Nanoparticles Alleviate Neuroinflammation and Neurotoxicity in a Rat Model of Alzheimer's Disease by Regulating Sirt1/miRNA-134/GSK3β Expression. *Biol Trace Elem Res* 200(12)**,** 5104-5114. doi: 10.1007/s12011-021-03073-7.

Abuelella, K.E., Abd-Allah, H., Soliman, S.M., and Abdel-Mottaleb, M.M.A. (2023). Skin targeting by chitosan/hyaluronate hybrid nanoparticles for the management of irritant contact dermatitis: In vivo therapeutic efficiency in mouse-ear dermatitis model. *Int J Biol Macromol* 232**,** 123458. doi: 10.1016/j.ijbiomac.2023.123458.

Aldawsari, M.F., Alalaiwe, A., Khafagy, E.S., Al Saqr, A., Alshahrani, S.M., Alsulays, B.B., et al. (2021). Efficacy of SPG-ODN 1826 Nanovehicles in Inducing M1 Phenotype through TLR-9 Activation in Murine Alveolar J774A.1 Cells: Plausible Nano-Immunotherapy for Lung Carcinoma. *Int J Mol Sci* 22(13). doi: 10.3390/ijms22136833.

Alomari, G., Al-Trad, B., Hamdan, S., Aljabali, A., Al-Zoubi, M., Bataineh, N., et al. (2020). Gold nanoparticles attenuate albuminuria by inhibiting podocyte injury in a rat model of diabetic nephropathy. *Drug delivery and translational research* 10**,** 216-226.

Arif, M., Ahmad, R., Sharaf, M., Muhammad, J., Abdalla, M., Eltayb, W.A., et al. (2022). Antibacterial and antibiofilm activity of mannose-modified chitosan/PMLA nanoparticles against multidrug-resistant Helicobacter pylori. *International Journal of Biological Macromolecules* 223**,** 418-432.

Asad, M.I., Khan, D., Rehman, A.U., Elaissari, A., and Ahmed, N. (2021). Development and in vitro/in vivo evaluation of pH-sensitive polymeric nanoparticles loaded hydrogel for the management of psoriasis. *Nanomaterials* 11(12)**,** 3433.

Bashir, D.J., Manzoor, S., Khan, I.A., Bashir, M., Agarwal, N.B., Rastogi, S., et al. (2022). Nanonization of Magnoflorine-Encapsulated Novel Chitosan-Collagen Nanocapsules for Neurodegenerative Diseases: In Vitro Evaluation. *ACS Omega* 7(8)**,** 6472-6480. doi: 10.1021/acsomega.1c04459.

Bashir, D.J., Manzoor, S., Sarfaraj, M., Afzal, S.M., Bashir, M., Nidhi, et al. (2023). Magnoflorine-Loaded Chitosan Collagen Nanocapsules Ameliorate Cognitive Deficit in Scopolamine-Induced Alzheimer’s Disease-like Conditions in a Rat Model by Downregulating IL-1β, IL-6, TNF-α, and Oxidative Stress and Upregulating Brain-Derived Neurotrophic Factor and DCX Expressions. *ACS Omega*.

Bilia, A.R., Bergonzi, M.C., Isacchi, B., Antiga, E., and Caproni, M. (2018). Curcumin nanoparticles potentiate therapeutic effectiveness of acitrein in moderate-to-severe psoriasis patients and control serum cholesterol levels. *J Pharm Pharmacol* 70(7)**,** 919-928. doi: 10.1111/jphp.12910.

Cheng, G., Liu, X., Liu, Y., Liu, Y., Ma, R., Luo, J., et al. (2022). Ultrasmall coordination polymers for alleviating ROS-mediated inflammatory and realizing neuroprotection against Parkinson’s disease. *Research* 2022.

Chiu, Y.-H., Wu, Y.-W., Hung, J.-I., and Chen, M.-C. (2021). Epigallocatechin gallate/L-ascorbic acid–loaded poly-γ-glutamate microneedles with antioxidant, anti-inflammatory, and immunomodulatory effects for the treatment of atopic dermatitis. *Acta Biomaterialia* 130**,** 223-233.

Cortial, A., Nosbaum, A., Rozières, A., Baeck, M., de Montjoye, L., Grande, S., et al. (2015). Encapsulation of hydrophobic allergens into nanoparticles improves the in vitro immunological diagnosis of allergic contact dermatitis. *Nanomedicine* 11(4)**,** 1029-1033. doi: 10.1016/j.nano.2015.02.001.

Cui, Y., Zhang, J., Liu, Y., Meng, G., and Lv, C. (2022). Engineering Mucic Acid Loaded Polyethylenimine@ GoldNanoparticles for Improving the Treatment of Rheumatoid Arthritis. *Journal of Cluster Science* 33(6)**,** 2419-2427.

Di Mauro, G., Amoriello, R., Lozano, N., Carnasciali, A., Guasti, D., Becucci, M., et al. (2023). Graphene Oxide Nanosheets Reduce Astrocyte Reactivity to Inflammation and Ameliorate Experimental Autoimmune Encephalomyelitis. *ACS nano* 17(3)**,** 1965-1978.

Elmowafy, E., El-Gogary, R.I., Ragai, M.H., and Nasr, M. (2019). Novel antipsoriatic fluidized spanlastic nanovesicles: In vitro physicochemical characterization, ex vivo cutaneous retention and exploratory clinical therapeutic efficacy. *Int J Pharm* 568**,** 118556. doi: 10.1016/j.ijpharm.2019.118556.

Fu, X., Yu, X., Jiang, J., Yang, J., Chen, L., Yang, Z., et al. (2022). Small molecule-assisted assembly of multifunctional ceria nanozymes for synergistic treatment of atherosclerosis. *Nature Communications* 13(1)**,** 6528.

Gu, Z., Li, F., Liu, Y., Jiang, M., Zhang, L., He, L., et al. (2021). Exosome‐Like Nanoparticles From Lactobacillus rhamnosus GG Protect Against Alcohol‐Associated Liver Disease Through Intestinal Aryl Hydrocarbon Receptor in Mice. *Hepatology Communications* 5(5)**,** 846-864.

Guo, Q., Chen, C., Wu, Z., Zhang, W., Wang, L., Yu, J., et al. (2022a). Engineered PD-1/TIGIT dual-activating cell-membrane nanoparticles with dexamethasone act synergistically to shape the effector T cell/Treg balance and alleviate systemic lupus erythematosus. *Biomaterials* 285**,** 121517.

Guo, X., Xu, L., Velazquez, H., Chen, T.-M., Williams, R.M., Heller, D.A., et al. (2022b). Kidney-targeted renalase agonist prevents cisplatin-induced chronic kidney disease by inhibiting regulated necrosis and inflammation. *Journal of the American Society of Nephrology* 33(2)**,** 342-356.

Hao, X., Zhang, H., Liu, R., Che, J., Zhang, D., Liang, J., et al. (2022). Red blood cell membrane functionalized biomimetic nanoparticles for systemic lupus erythematosus treatment. *Materials Today Advances* 16**,** 100294.

He, Y., de Araújo Júnior, R.F., Cavalcante, R.S., Yu, Z., Schomann, T., Gu, Z., et al. (2023). Effective breast cancer therapy based on palmitic acid-loaded PLGA nanoparticles. *Biomater Adv* 145**,** 213270. doi: 10.1016/j.bioadv.2022.213270.

Huang, L., Zhang, X., Ding, Z., Qi, Y., Wang, W., Xu, X., et al. (2022). PEGylated 2D-nanomaterials alleviate Parkinson's disease by shielding PIP2 lipids to inhibit IP3 second messenger signaling. *Nano Today* 46**,** 101556.

Huynh, P.T., Nguyen, G.D., Tran, K.T.L., Ho, T.M., Duong, B.T., Lam, V.Q., et al. (2021). One-pot, surfactant-free synthesis of gold nanostars and evaluation of their antibacterial effects against propionibacterium acnes. *Journal of Nanomaterials* 2021**,** 1-10.

Jiang, B., Zhang, Y., Li, Y., Chen, Y., Sha, S., Zhao, L., et al. (2022). A Tissue-Tended Mycophenolate-Modified Nanoparticle Alleviates Systemic Lupus Erythematosus in MRL/Lpr Mouse Model Mainly by Promoting Local M2-Like Macrophagocytes Polarization. *Int J Nanomedicine* 17**,** 3251-3267. doi: 10.2147/ijn.S361400.

Jin, M., Li, N., Sheng, W., Ji, X., Liang, X., Kong, B., et al. (2021a). Toxicity of different zinc oxide nanomaterials and dose-dependent onset and development of Parkinson’s disease-like symptoms induced by zinc oxide nanorods. *Environment International* 146**,** 106179.

Jin, M., Li, S., Wu, Y., Li, D., and Han, Y. (2021b). Construction of chitosan/alginate nano-drug delivery system for improving dextran sodium sulfate-induced colitis in mice. *Nanomaterials* 11(8)**,** 1884.

Jing, Q., Ruan, H., Li, J., Wang, Z., Pei, L., Hu, H., et al. (2021). Keratinocyte membrane-mediated nanodelivery system with dissolving microneedles for targeted therapy of skin diseases. *Biomaterials* 278**,** 121142.

Khater, S.I., Mohamed, A.A., Arisha, A.H., Ebraheim, L.L.M., El-Mandrawy, S.A.M., Nassan, M.A., et al. (2021). Stabilized-chitosan selenium nanoparticles efficiently reduce renal tissue injury and regulate the expression pattern of aldose reductase in the diabetic-nephropathy rat model. *Life Sci* 279**,** 119674. doi: 10.1016/j.lfs.2021.119674.

Kim, C.S., Mathew, A.P., Vasukutty, A., Uthaman, S., Joo, S.Y., Bae, E.H., et al. (2021). Glycol chitosan-based tacrolimus-loaded nanomicelle therapy ameliorates lupus nephritis. *J Nanobiotechnology* 19(1)**,** 109. doi: 10.1186/s12951-021-00857-w.

Kim, N.G., Jung, D.J., Jung, Y.K., and Kang, K.S. (2023). The Effect of a Novel Mica Nanoparticle, STB-MP, on an Alzheimer's Disease Patient-Induced PSC-Derived Cortical Brain Organoid Model. *Nanomaterials (Basel)* 13(5). doi: 10.3390/nano13050893.

Lai, X., Wang, M., Zhu, Y., Feng, X., Liang, H., Wu, J., et al. (2021). ZnO NPs delay the recovery of psoriasis-like skin lesions through promoting nuclear translocation of p-NFκB p65 and cysteine deficiency in keratinocytes. *Journal of hazardous materials* 410**,** 124566.

Lee, K.-J., Ratih, K., Kim, G.-J., Lee, Y.-R., Shin, J.-S., Chung, K.-H., et al. (2022). Immunomodulatory and anti-inflammatory efficacy of hederagenin-coated maghemite (γ-Fe2O3) nanoparticles in an atopic dermatitis model. *Colloids and Surfaces B: Biointerfaces* 210**,** 112244.

Li, H., Zhu, J., Xu, Y.-w., Mou, F.-f., Shan, X.-l., Wang, Q.-l., et al. (2022a). Notoginsenoside R1-loaded mesoporous silica nanoparticles targeting the site of injury through inflammatory cells improves heart repair after myocardial infarction. *Redox Biology* 54**,** 102384.

Li, M., Wang, Y., Han, X., Liu, Y., Ma, M., and Zhang, L. (2022b). Multifunctional Polydopamine-Based Nanoparticles for Dual-Mode Imaging Guided Targeted Therapy of Lupus Nephritis. *Pharmaceutics* 14(10)**,** 1988.

Li, Y., Chen, X., Jin, R., Chen, L., Dang, M., Cao, H., et al. (2021). Injectable hydrogel with MSNs/microRNA-21-5p delivery enables both immunomodification and enhanced angiogenesis for myocardial infarction therapy in pigs. *Science advances* 7(9)**,** eabd6740.

Lin, Y., Yi, O., Hu, M., Hu, S., Su, Z., Liao, J., et al. (2022). Multifunctional nanoparticles of sinomenine hydrochloride for treat-to-target therapy of rheumatoid arthritis via modulation of proinflammatory cytokines. *Journal of Controlled Release* 348**,** 42-56.

Liu, S., Xu, A., Gao, Y., Xie, Y., Liu, Z., Sun, M., et al. (2021). Graphene oxide exacerbates dextran sodium sulfate-induced colitis via ROS/AMPK/p53 signaling to mediate apoptosis. *Journal of Nanobiotechnology* 19(1)**,** 1-15.

Liu, Y., Meng, C., Li, Y., Xia, D., Lu, C., Lai, J., et al. (2023). Peptide-Protected Gold Nanoclusters Efficiently Ameliorate Acute Contact Dermatitis and Psoriasis via Repressing the TNF-α/NF-κB/IL-17A Axis in Keratinocytes. *Nanomaterials* 13(4)**,** 662.

Lu, L., Qi, S., Chen, Y., Luo, H., Huang, S., Yu, X., et al. (2020). Targeted immunomodulation of inflammatory monocytes across the blood-brain barrier by curcumin-loaded nanoparticles delays the progression of experimental autoimmune encephalomyelitis. *Biomaterials* 245**,** 119987. doi: 10.1016/j.biomaterials.2020.119987.

Lu, Z., Ma, L., Mei, L., Ren, K., Li, M., Zhang, L., et al. (2022). Micellar nanoparticles inhibit the postoperative inflammation, recurrence and pulmonary metastasis of 4T1 breast cancer by blocking NF-κB pathway and promoting MDSCs depletion. *Int J Pharm* 628**,** 122303. doi: 10.1016/j.ijpharm.2022.122303.

Ma, Q., Bian, L., Zhao, X., Tian, X., Yin, H., Wang, Y., et al. (2022). Novel glucose-responsive nanoparticles based on p-hydroxyphenethyl anisate and 3-acrylamidophenylboronic acid reduce blood glucose and ameliorate diabetic nephropathy. *Materials Today Bio* 13**,** 100181.

Mangal, J.L., Inamdar, S., Suresh, A.P., Jaggarapu, M., Esrafili, A., Ng, N.D., et al. (2022). Short term, low dose alpha-ketoglutarate based polymeric nanoparticles with methotrexate reverse rheumatoid arthritis symptoms in mice and modulate T helper cell responses. *Biomater Sci* 10(23)**,** 6688-6697. doi: 10.1039/d2bm00415a.

Manna, K., Mishra, S., Saha, M., Mahapatra, S., Saha, C., Yenge, G., et al. (2019). Amelioration of diabetic nephropathy using pomegranate peel extract-stabilized gold nanoparticles: assessment of NF-κB and Nrf2 signaling system. *Int J Nanomedicine* 14**,** 1753-1777. doi: 10.2147/ijn.S176013.

Meng, T., Jiang, R., Wang, S., Li, J., Zhang, F., Lee, J.-H., et al. (2020). Stem cell membrane-coated Au-Ag-PDA nanoparticle-guided photothermal acne therapy. *Colloids and Surfaces B: Biointerfaces* 192**,** 111145.

Midander, K., Werner, P., Isaksson, M., Wisgrill, L., Lidén, C., Fyhrquist, N., et al. (2023). Cobalt nanoparticles cause allergic contact dermatitis in humans. *British Journal of Dermatology* 188(2)**,** 278-287.

Nag, S., Manna, K., Saha, M., and Das Saha, K. (2020). Tannic acid and vitamin E loaded PLGA nanoparticles ameliorate hepatic injury in a chronic alcoholic liver damage model via EGFR-AKT-STAT3 pathway. *Nanomedicine* 15(3)**,** 235-257.

Pinto, B.F., Ribeiro, L.N.B., da Silva, G., Freitas, C.S., Kraemer, L., Oliveira, F.M.S., et al. (2022). Inhalation of dimethyl fumarate-encapsulated solid lipid nanoparticles attenuate clinical signs of experimental autoimmune encephalomyelitis and pulmonary inflammatory dysfunction in mice. *Clin Sci (Lond)* 136(1)**,** 81-101. doi: 10.1042/cs20210792.

Poon, C., Gallo, J., Joo, J., Chang, T., Bañobre-López, M., and Chung, E.J. (2018). Hybrid, metal oxide-peptide amphiphile micelles for molecular magnetic resonance imaging of atherosclerosis. *J Nanobiotechnology* 16(1)**,** 92. doi: 10.1186/s12951-018-0420-8.

Radwan, E., Ali, M., Faied, S.M.A., Omar, H.M., Mohamed, W.S., Abd-Elghaffar, S.K., et al. (2021). Novel therapeutic regimens for urethane-induced early lung cancer in rats: Combined cisplatin nanoparticles with vitamin-D(3). *IUBMB Life* 73(2)**,** 362-374. doi: 10.1002/iub.2432.

Rouco, H., Diaz-Rodriguez, P., Gaspar, D.P., Gonçalves, L.M., Cuerva, M., Remuñán-López, C., et al. (2020). Rifabutin-loaded nanostructured lipid carriers as a tool in oral anti-mycobacterial treatment of Crohn’s disease. *Nanomaterials* 10(11)**,** 2138.

Salimi, M., Sarkar, S., Hashemi, M., and Saber, R. (2020). Treatment of Breast Cancer-Bearing BALB/c Mice with Magnetic Hyperthermia using Dendrimer Functionalized Iron-Oxide Nanoparticles. *Nanomaterials* 10(11)**,** 2310.

Schwarzfischer, M., Niechcial, A., Handler, K., Morsy, Y., Wawrzyniak, M., Laimbacher, A.S., et al. (2022). TiO2 nanoparticles abrogate the protective effect of the Crohn’s disease-associated variation within the PTPN22 gene locus. *Gut*.

Shi, C., Dawulieti, J., Shi, F., Yang, C., Qin, Q., Shi, T., et al. (2022). A nanoparticulate dual scavenger for targeted therapy of inflammatory bowel disease. *Science Advances* 8(4)**,** eabj2372.

Siddique, M.I., Katas, H., Jamil, A., Mohd Amin, M.C.I., Ng, S.F., Zulfakar, M.H., et al. (2019). Potential treatment of atopic dermatitis: tolerability and safety of cream containing nanoparticles loaded with hydrocortisone and hydroxytyrosol in human subjects. *Drug Deliv Transl Res* 9(2)**,** 469-481. doi: 10.1007/s13346-017-0439-7.

Stachyra, K., Wiśniewska, A., Kiepura, A., Kuś, K., Rolski, F., Czepiel, K., et al. (2022). Inhaled silica nanoparticles exacerbate atherosclerosis through skewing macrophage polarization towards M1 phenotype. *Ecotoxicology and Environmental Safety* 230**,** 113112.

Sun, T., Kwong, C.H., Gao, C., Wei, J., Yue, L., Zhang, J., et al. (2020). Amelioration of ulcerative colitis via inflammatory regulation by macrophage-biomimetic nanomedicine. *Theranostics* 10(22)**,** 10106.

Suresh, P., Salem-Bekhit, M.M., Veedu, H.P., Alshehri, S., Nair, S.C., Bukhari, S.I., et al. (2022). Development of a Novel Methotrexate-Loaded Nanoemulsion for Rheumatoid Arthritis Treatment with Site-Specific Targeting Subcutaneous Delivery. *Nanomaterials* 12(8)**,** 1299.

Uhlířová, R., Langová, D., Bendová, A., Gross, M., Skoumalová, P., and Márová, I. (2023). Antimicrobial Activity of Gelatin Nanofibers Enriched by Essential Oils against Cutibacterium acnes and Staphylococcus epidermidis. *Nanomaterials* 13(5)**,** 844.

Wang, Q., Wang, Y., Liu, S., Sha, X., Song, X., Dai, Y., et al. (2021a). Theranostic nanoplatform to target macrophages enables the inhibition of atherosclerosis progression and fluorescence imaging of plaque in ApoE (−/−) mice. *Journal of Nanobiotechnology* 19(1)**,** 1-17.

Wang, Z., Wang, H., Cheng, X., Geng, J., Wang, L., Dong, Q., et al. (2021b). Aptamer-superparamagnetic nanoparticles capture coupling siderophore-Fe3+ scavenging actuated with carbon dots to confer an “off-on” mechanism for the ultrasensitive detection of Helicobacter pylori. *Biosensors and Bioelectronics* 193**,** 113551.

Wen, L., Li, M., Lin, X., Li, Y., Song, H., and Chen, H. (2022). AgNPs Aggravated Hepatic Steatosis, Inflammation, Oxidative Stress, and Epigenetic Changes in Mice With NAFLD Induced by HFD. *Front Bioeng Biotechnol* 10**,** 912178. doi: 10.3389/fbioe.2022.912178.

Yahya, R., Al-Rajhi, A.M., Alzaid, S.Z., Al Abboud, M.A., Almuhayawi, M.S., Al Jaouni, S.K., et al. (2022). Molecular docking and efficacy of Aloe vera gel based on chitosan nanoparticles against Helicobacter pylori and its antioxidant and anti-inflammatory activities. *Polymers* 14(15)**,** 2994.

You, P., Mayier, A., Zhou, H., Yang, A., Fan, J., Ma, S., et al. (2022). Targeting and promoting atherosclerosis regression using hybrid membrane coated nanomaterials via alleviated inflammation and enhanced autophagy. *Applied Materials Today* 26**,** 101386.

Yuan, Z., Qin, Q., Yuan, M., Wang, H., and Li, R. (2020). Development and novel design of clustery graphene oxide formed Conductive Silk hydrogel cell vesicle to repair and routine care of myocardial infarction: Investigation of its biological activity for cell delivery applications. *Journal of Drug Delivery Science and Technology* 60**,** 102001.

Zampieri, R., Brozzetti, A., Pericolini, E., Bartoloni, E., Gabrielli, E., Roselletti, E., et al. (2020). Prevention and treatment of autoimmune diseases with plant virus nanoparticles. *Sci Adv* 6(19)**,** eaaz0295. doi: 10.1126/sciadv.aaz0295.

Zelepukin, I.V., Ivanov, I.N., Mirkasymov, A.B., Shevchenko, K.G., Popov, A.A., Prasad, P.N., et al. (2022). Polymer-coated BiOCl nanosheets for safe and regioselective gastrointestinal X-ray imaging. *J Control Release* 349**,** 475-485. doi: 10.1016/j.jconrel.2022.07.007.

Zhang, J., Guo, Q., Dai, D., Yu, J., Wang, L., Wu, Z., et al. (2022a). Rapamycin-encapsulated costimulatory ICOS/CD40L-bispecific nanoparticles restrict pathogenic helper TB-cell interactions while in situ suppressing mTOR for lupus treatment. *Biomaterials* 289**,** 121766.

Zhang, J., Li, X., Cheng, W., Li, Y., Shi, T., Jiang, Y., et al. (2022b). Chronic carbon black nanoparticles exposure increases lung cancer risk by affecting the cell cycle via circulatory inflammation. *Environmental Pollution* 305**,** 119293.

Zhang, L., Cheng, M., Lin, Y., Zhang, J., Shen, B., Chen, Y., et al. (2022c). Ultrasound-assisted carbon nanoparticle suspension mapping versus dual tracer-guided sentinel lymph node biopsy in patients with early breast cancer (ultraCars): phase III randomized clinical trial. *Br J Surg* 109(12)**,** 1232-1238. doi: 10.1093/bjs/znac311.

Zhang, T., Dang, M., Zhang, W., and Lin, X. (2020). Gold nanoparticles synthesized from Euphorbia fischeriana root by green route method alleviates the isoprenaline hydrochloride induced myocardial infarction in rats. *Journal of Photochemistry and Photobiology B: Biology* 202**,** 111705.

Zhang, W., Zhou, Y., Fan, Y., Cao, R., Xu, Y., Weng, Z., et al. (2022d). Metal–Organic‐Framework‐Based Hydrogen‐Release Platform for Multieffective Helicobacter Pylori Targeting Therapy and Intestinal Flora Protective Capabilities. *Advanced Materials* 34(2)**,** 2105738.

Zhao, R., Zhu, M., Zhou, S., Feng, W., and Chen, H. (2020). Rapamycin-loaded mPEG-PLGA nanoparticles ameliorate hepatic steatosis and liver injury in non-alcoholic fatty liver disease. *Frontiers in Chemistry* 8**,** 407.

Zhu, M., Niu, Q., Zhang, J., Yu, Y., Wang, H., Zhu, T., et al. (2022). Amorphous selenium nanodots alleviate non-alcoholic fatty liver disease via activating VEGF receptor 1 to further inhibit phosphorylation of JNK/p38 MAPK pathways. *Eur J Pharmacol* 932**,** 175235. doi: 10.1016/j.ejphar.2022.175235.
